# Relaxase MobM Induces a Molecular Switch at Its Cognate Origin of Transfer

**DOI:** 10.3389/fmolb.2018.00017

**Published:** 2018-02-26

**Authors:** Fabián Lorenzo-Díaz, Cris Fernández-López, Beatriz Guillén-Guío, Alicia Bravo, Manuel Espinosa

**Affiliations:** ^1^Departamento de Bioquímica, Microbiología, Biología Celular y Genética, Universidad de La Laguna, Santa Cruz de Tenerife, Spain; ^2^Unidad de Investigación, Hospital Universitario Nuestra Señora de Candelaria, Santa Cruz de Tenerife, Spain; ^3^Centro de Investigaciones Biológicas, CSIC, Madrid, Spain

**Keywords:** MobM relaxase, plasmid pMV158, DNA-protein interactions, hairpin formation, origin of transfer

## Abstract

The MOB_V1_ family of relaxases is broadly distributed in plasmids and other mobile genetic elements isolated from staphylococci, enterococci, and streptococci. The prototype of this family is protein MobM encoded by the streptococcal promiscuous plasmid pMV158. MobM cleaves the phosphodiester bond of a specific dinucleotide within the origin of transfer (*oriT*) to initiate conjugative transfer. Differently from other relaxases, MobM and probably other members of the family, cleaves its target single-stranded DNA through a histidine residue rather than the commonly used tyrosine. The *oriT* of the MOB_V1_ family differs from other well-known conjugative systems since it has sequences with three inverted repeats, which were predicted to generate three mutually-exclusive hairpins on supercoiled DNA. In this work, such hypothesis was evaluated through footprinting experiments on supercoiled plasmid DNA. We have found a change in hairpin extrusion mediated by protein MobM. This conformational change involves a shift from the main hairpin generated on “naked” DNA to a different hairpin in which the nick site is positioned in a single-stranded configuration. Our results indicate that the *oriT*_pMV158_ acts as a molecular switch in which, depending on the inverted repeat recognized by MobM, pMV158 mobilization could be turned “on” or “off.”

## Introduction

Horizontal Gene Transfer (HGT) is the main source to acquire novel gene traits by organisms. It is mediated by plasmids and other Mobile Genetic Elements (MGE) that use conjugation as the most frequent process to perform DNA transfer (de la Cruz and Davies, 2000). Conjugation involves physical contact between a donor and a recipient cell, and the process is initiated and terminated by dedicated topoisomerase endonuclease-like proteins termed relaxases (de la Cruz and Davies, 2000; Chandler et al., 2013; Lorenzo-Díaz et al., 2016). Conjugative DNA transfer is not restricted to bacteria, since trans-kingdom exchange of DNA is known (González-Prieto et al., 2013), and the number of instances of HGT between bacteria and eukaryotes is increasing (Lacroix and Citovsky, 2016). Conjugation is mediated by a relaxase that initiates (in the donor) and terminates (in the recipient) the transfer by binding to a strand- and sequence-specific region (the origin of transfer, *oriT*) followed by cleavage of the phosphodiester bond at a specific dinucleotide, generating a covalent protein-DNA complex termed relaxosome (de la Cruz et al., 2010; Chandler et al., 2013). The cleavage reaction generates a covalent amino acyl-DNA adduct that is pumped from donor to recipient cells (Llosa et al., 2002) through a plasmid-encoded multiprotein complex that is composed by the coupling protein and a Type-IV secretion system (Goessweiner-Mohr et al., 2013; Low et al., 2014; Ilangovan et al., 2017; Trokter and Waksman, 2018).

**Figure 1:**
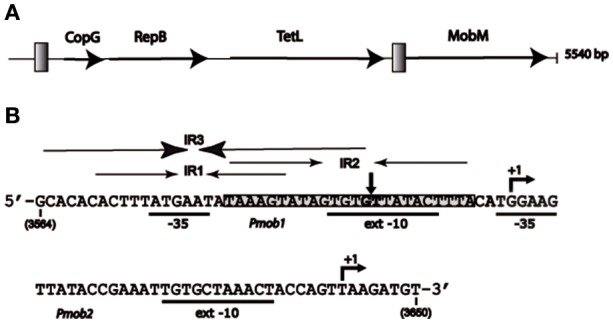
Representation of plasmid pMV158 and its *oriT* region. **(A)** Schematic map of the plasmid showing the proteins that it encodes: transcriptional repressor CopG, replicase RepB, tetracycline resistance determinant type L (TetL), and relaxase MobM. Gray squares represent the two regions in which the main inverted repeat (IR) sequences have been located. **(B)** Nucleotide sequence of the pMV158 region spanning coordinates 3564 and 3650. This region includes *oriT*_pMV158_ and the two promoters of the *mobM* gene (*Pmob1* and *Pmob2*) with the −35 and extended −10 boxes underlined. The transcription start sites of *mobM* (+1 position) are indicated. The three overlapping inverted repeats (IR1, IR2, and IR3), and the nick site (vertical arrow between G3595 and T3596) are indicated. The shadowed sequence (coordinates 3582–3605) indicates the MobM binding site on linear double-stranded DNA (dsDNA) defined by DNase I footprinting assays (Grohmann et al., 1999).

MGE have been classified on the basis of their ability to encode all elements needed to transfer (conjugative) or only the relaxase protein (mobilizable). Within this latter category, there is a class of small plasmids that replicate by the rolling-circle mechanism, and thus termed RCR-plasmids (Novick, 1998; Khan, 2005; Espinosa, 2013; Ruiz-Masó et al., 2015). These plasmids are very abundant (hundreds of them reported so far), participate actively in the spread of antibiotic resistance traits, and may encode up to two DNA-relaxing proteins, the replicase and the relaxase, involved in replication and mobilization, respectively. The DNA substrate of these proteins is either supercoiled (sc) or single-stranded (ss) DNA, because the dinucleotide to be cleaved must be exposed in a single-stranded configuration (Espinosa, 2013). Most plasmids exhibit negative DNA supercoiling, which produces torsional stresses that are released by generation of stem-loop structures at inverted repeats (Lilley, 1980). Supercoiling influences many biological processes, replication and transcription among them (Stillman and Gluzman, 1985; Liu and Wang, 1987). In the case of pMV158, influence of the degree of supercoiling on the recognition and cleavage at the plasmid replication origin by its cognate RepB replicase has been previously shown (Moscoso et al., 1995). Gene *repB* is co-transcribed with the gene *copG*, which encodes the transcriptional repressor protein, CopG (see Figure 1); this small protein (45 amino acids per protomer) regulates its own synthesis and the synthesis of the replicase RepB, thus limiting the intracellular amounts of the initiator of replication protein (del Solar et al., 2002).

In addition to the RepB replicase, pMV158 encodes the MobM relaxase (Guzmán and Espinosa, 1997). Protein MobM is the prototype of the MOB_V1_ superfamily of relaxases (Francia et al., 2004; Garcillán-Barcia et al., 2009). Recently, MobM was shown to exhibit a distinct mechanism for DNA nicking/closing, which is mediated by a histidine residue that generates a protein-DNA phosphoramidate adduct rather than the commonly used tyrosine-mediated DNA cleavage (Chandler et al., 2013; Pluta et al., 2017). The MOB_V1_ family of relaxases has more than 100 members (Lorenzo-Díaz et al., 2014), and it is especially populated in low G+C Firmicutes, notably staphylococci, streptococci, and enterococci (Fernández-López et al., 2014; Pluta et al., 2017). Like most mobilizable RCR-plasmids, pMV158 lacks the elements required to be self-transmissible except the MobM relaxase. Thus, to be transferred, pMV158 uses the functions provided by auxiliary plasmids (Priebe and Lacks, 1989; Farías and Espinosa, 2000). MobM initiates transfer by cleaving the 5′-GpT-3′ phosphodiester bond (coordinates 3595–3596 in the pMV158 sequence) (Guzmán and Espinosa, 1997). The full length MobM is a dimer of 57.9 kDa per protomer (de Antonio et al., 2004) that is able to repress the transcription of its own gene by binding to a region that contains sequence elements of two promoters (*Pmob1* and *Pmob2*) as well as sequences of the origin of transfer, *oriT*_pMV158_ (Lorenzo-Díaz et al., 2012). To cleave, but not to bind to, its target DNA, MobM requires scDNA or ssDNA as substrate, suggesting that the dinucleotide to be cleaved (the nick site) should be located within an unpaired region of a plasmid hairpin (Lorenzo-Díaz et al., 2011). The region encompassing the *oriT*_pMV158_ (see Figure 1) contains three inverted repeats (IRs) that could generate three mutually exclusive hairpin structures on scDNA (Lorenzo-Díaz et al., 2011). Such a region contains the *Pmob1* promoter and is adjacent to the *Pmob2* promoter (Lorenzo-Díaz et al., 2012). In the present work, we have addressed the interactions between MobM and its target scDNA by identification of the footprints left by the protein at its cognate *oriT*_pMV158_. We show that MobM promotes conformational changes in the *oriT*_pMV158_ that lead to a shift in hairpin extrusion. We propose a dynamic model in which a hairpin formed on “naked” DNA changes its conformation upon MobM binding. This would result in a local DNA melting and exposure of the nick site in ssDNA configuration to generate the substrate that is suitable for the initiation of conjugation. The region encompassing the *oriT*_pMV158_ was found to be present in many bacterial genomes, making our findings more widespread than expected.

## Materials and methods

### Materials

Tryptone yeast extract (TY) culture medium was acquired from Pronadisa (Spain). Tris, MgCl_2_, CaCl_2_, NaCl, EDTA, lysozyme, SDS, and dithiothreitol (DTT) were obtained from Fisher Scientific. Antibiotics, CsCl, sucrose, yeast extract and the reagents for the footprinting assays were of the highest quality and purchased to Merck, Sigma, or BioRad. [γ-^32^P]-ATP was from PerkinElmer. Protease inhibitor cocktail was from Roche. Agarose, heparin-agarose, and Superdex were from BioRad Laboratories. Other enzymes were from New England Biolabs.

### Strains, plasmids, and protein purification

*Escherichia coli* cells were grown in TY medium, which was supplemented with ampicillin (100 μg/ml) when they harbored plasmid pLGM2 (Guzmán and Espinosa, 1997). This plasmid is a pET5 derivative that carries the *mobM* gene under control of the Φ10 promoter of phage T7. *E. coli* BL21(DE3) (a gift of F. W. Studier) was used for overproduction of MobM (Lorenzo-Díaz et al., 2011). *Streptococcus pneumoniae* 708 (*trt-1, hex-4, end-1, exo-2, malM594*) was used as the host for plasmid pMV158 (GenBank NC_010096). This plasmid was isolated from *Streptococcus agalactiae* (Burdett, 1980) and carries a constitutive tetracycline resistance determinant of the *tetL* category. *S. pneumoniae* cells harboring pMV158 were grown in AGCH medium (Ruiz-Cruz et al., 2010), supplemented with 0.2% yeast extract, 0.3% sucrose, and 1 μg/ml tetracycline. Supercoiled pMV158 DNA was purified from *S. pneumoniae* 708 by two consecutive CsCl gradients (del Solar et al., 1987).

Native full-length MobM protein was overproduced, purified and stored as previously reported (Lorenzo-Díaz et al., 2011). Briefly, the protein was purified from 4 l of TY medium from *E. coli* cultures after induction with 1 mM IPTG (30 min) and further incubation with rifampicin (200 μg/ml, 90 min). Cells were concentrated 100-fold, resuspended in buffer A (20 mM Tris–HCl pH 7.6, 1 mM EDTA, 1 mM dithiothreitol, 5% (v/v) glycerol) containing 1 M NaCl and a tablet of protease inhibitor cocktail, and lysed by passage through a French pressure cell. Nucleic acids in the cell extract were precipitated with 0.2% (v/v) polyethyleneimine, and proteins in the supernatant were precipitated at 70% (w/v) ammonium sulfate saturation. Proteins in the precipitate were dissolved in buffer A with 500 mM NaCl and purified by two chromatographic steps (heparin–agarose, and HiLoad Superdex 200 gel-filtration). Fractions containing pure protein (>98%) were pooled, concentrated until 5 mg/ml protein, and stored at −80°C. In these conditions, the protein retained full activity for at least 1 year. Edman's sequential degradation was used to determine the first 10 residues of the N-terminal sequence of the purified MobM, and its concentration was calculated by spectrophotometry and by determination of their amino acid composition.

### Footprinting assays on supercoiled plasmid DNA

Binding reactions (45 μl) contained 20 mM Tris-HCl, pH 7.6, 1 mM EDTA, 1 mM DTT, 1% glycerol, 50 mM NaCl, MobM (0.25, 0.5, 1, or 2 μM), and pMV158 scDNA (2 μg). Reaction mixtures were incubated at 25°C for 25 min. Then, for DNase I footprinting assays, 5 μl of a solution that contained 0.5 units of DNase I, 10 mM MgCl_2_ and 5 mM CaCl_2_ were added as reported (Lorenzo-Díaz et al., 2017). After 1 min at 25°C, DNase I digestion was stopped by adding 100 μl of stop buffer (1% SDS, 200 mM NaCl, 20 mM EDTA, pH 8.0). Under these conditions, a series of fragments that contain random 5′ and 3′ ends are generated. For dimethyl sulfate (DMS) footprinting assays, 5 μl of DMS (300 mM in 10 mM Tris-HCl, pH 8.0) were added to the binding reactions. After 5 min at 25°C, 50 μl of stop solution (20 mM EDTA, 3 M ammonium acetate, 1 M β-mercaptoethanol) were added. For KMnO_4_ footprinting assays, KMnO_4_ (5 mM final concentration) was added to the binding reactions. After 2 min at 25°C, 50 μl of stop solution (3 M β-mercaptoethanol, 40 mM EDTA, 0.6 M sodium acetate, pH 4.8) were added. In all the cases, DNA samples were extracted with phenol/chloroform, precipitated with ethanol, denatured with 0.2 M NaOH and neutralized. DNase I cleavage sites, as well as nucleotides modified by DMS or KMnO_4_, were mapped by primer extension using T7 DNA polymerase (Sequenase version) and a 5′-end radioactively labeled oligonucleotide, thus copying the hybridized strands to the 5′-end generated by the DNase I digestion or to the modified nucleotide as reported (Ruiz-Masó et al., 2007). Specifically, the F1 primer (5′- AACTGGTAGTTTAGCACAATTTCG-3′; coordinates 3643–3620 in pMV158, Accession Number NC_010096) or the PR2 primer (5′-GGTCGGCACTGCCGACAGC-3′; coordinates 3537–3555) were labeled at the 5′-end using [γ-^32^P]-ATP and T4 polynucleotide kinase. Samples were analyzed by sequencing gel (7 M urea, 8% polyacrylamide) electrophoresis. The loading buffer contained 80% formamide, 1 mM EDTA, 10 mM NaOH, 0.1% bromophenol blue, and 0.1% xylene cyanol. A sequence marker was obtained by sequencing plasmid pMV158 with the same ^32^P-labeled oligonucleotide (F1 or PR2).

### Bioinformatics analyses

Secondary structure predictions for the region spanning the *oriT* sequence were done with MFOLD software (Zuker, 2003). Densitometry profiles of DNA footprintings were performed with the Quantity One software (Bio-Rad). BLASTn analyses were preformed at the NCBI web site (https://blast.ncbi.nlm.nih.gov/Blast.cgi?PROGRAM=blastn&BLAST_PROGRAMS=megaBlast&PAGE_TYPE=BlastSearch) on October 1st, 2017. The parameters used in both programs were the default values.

## Results

### Predicted hairpin structures at the *oriT*_pMV158_

Plasmid pMV158 (5,540 bp) has four genes in its coding strand, namely those encoding the transcriptional repressor CopG, the RepB replicase, the tetracycline-resistance determinant, and the MobM relaxase (Espinosa, 2013; Figure 1A). The plasmid has two distinct regions in which sequences with IRs seem to accumulate: the region spanning the double-stranded origin of replication (Puyet et al., 1988), and the region spanning coordinates 3564–3606, which is located upstream of the *mobM* gene (Figure 1). The latter region includes the *oriT*_pMV158_ and the promoter *Pmob1*, and is adjacent to the promoter *Pmob2*. Both promoters direct transcription of *mobM* (Figure 1B; Farías et al., 1999; Lorenzo-Díaz et al., 2012). Overall, the region exhibits a high A+T content (72.1%) as compared to the entire plasmid (62.3%), indicative of flexible regions prone to melt.

**Figure 2:**
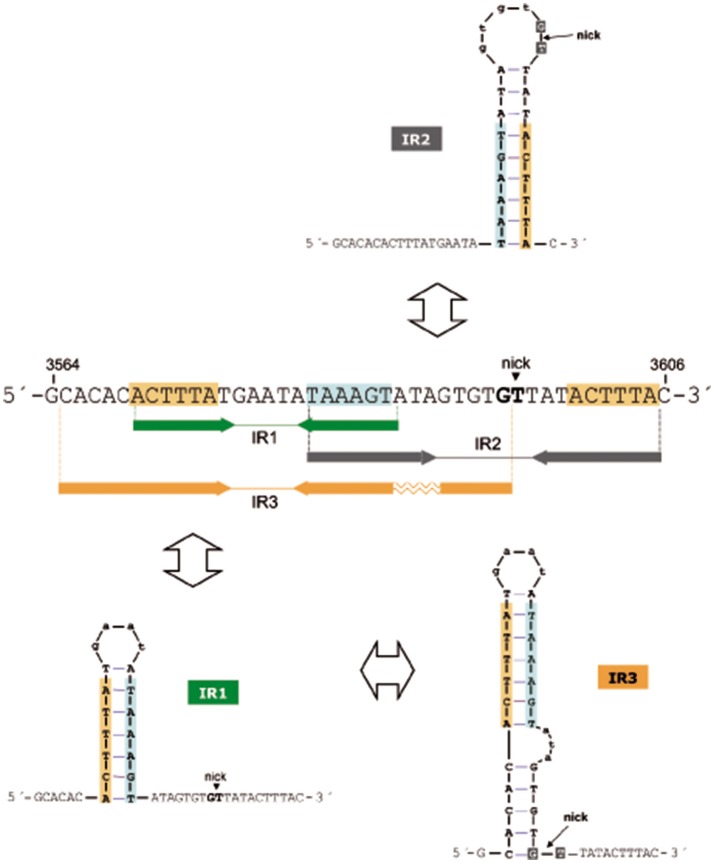
Possible hairpins generated at the *oriT*_pMV158_ are shown for the transferred DNA strand (coding strand). Sequence overlapping of the IRs suggests competition between them to generate cruciform structures on supercoiled plasmid DNA. Note that IR3 secondary structure formation/dissociation includes an IR1 intermediate. Horizontal arrows indicate sequences corresponding to the left and right arms of each IR. Sequence repetitions are depicted in colored background. The MobM-cleaved dinucleotide (5′-G/T-3′; nick site) is indicated.

The *oriT*_pMV158_ has three IRs (IR1, IR2, and IR3) that partially overlap (Lorenzo-Díaz et al., 2011), allowing the formation of three mutually exclusive stem-loop structures in which the specific dinucleotide cleaved by MobM (G3595/T3596, the nick site) would be placed in different positions (Figure 2). IR1 (predicted ΔG = −4.03 kcal/mol) spans 18 nt, and IR2 (predicted ΔG = −4.4 kcal/mol) has 24 nt; they could generate alternative stem-loop structures with 7- and 9-bp totally paired stems, respectively. Whereas IR1 is located 8 nt upstream of the nick site, the arms of IR2 encompass it. IR3 (predicted ΔG = −6.88 kcal/mol) includes the IR1 sequence and could extrude as a hairpin longer than IR1, with a 12-bp stem, a 4-nt loop, and an internal bulge-loop of 3 nt in the right arm (IR3-R). In IR3, the nick site is located just at the 3′-end of IR3-R (Figure 2). Furthermore, a directly repeated sequence (5′-ACTTTA-3′) is located at the IR1/IR3 left arm (IR1/IR3-L) and at the IR2 right arm (IR2-R). Since IR1/IR3-R and the left arm of IR2 (IR2-L) partially overlap, extrusion of IR2 would hinder extrusion of IR1/IR3 (and *vice versa*) on scDNA. Previous analyses (Lorenzo-Díaz et al., 2014) showed that the sequence that contains the three IRs is highly conserved among group A (42 members) of the nearly 100 members of the MOB_V1_ plasmid family (Lorenzo-Díaz et al., 2014; Supplementary Figure S1). These findings suggest that the IRs could be involved in the MobM-recognition of the *oriT*_pMV158_ at the initiation of transfer (relaxosome formation in the donor cell) and/or at the termination reaction to close the transferred DNA strand in the recipient cell.

The three-dimensional structures of some relaxases bound to their targets have shown that the nucleotide sequence of the *oriT* and its topological conformation are equally important. In the case of TrwC_R388_ and TraI_F_, both relaxases bind to oligonucleotides that mimic their cognate *oriT*s with higher affinity when they included a 5′-region corresponding to the IR located 8 nt and 9 nt upstream of the nick site, respectively (Guasch et al., 2003; Williams and Schildbach, 2006). Further, an oligonucleotide spanning the *oriT*_R388_ adopted a hairpin configuration when bound to the relaxase domain of TrwC_R388_ (Guasch et al., 2003; González-Pérez et al., 2007), and a similar situation was observed in the structure of the MobM relaxase domain bound to DNA (Pluta et al., 2017). Unlike other well-known origins of transfer, the configuration of the *oriT*_pMV158_ exhibits three IRs and one direct repeat around the nick site (Lorenzo-Díaz et al., 2014). Out of the three IRs, IR2 was predicted to expose the nick site in the loop of a cruciform structure (Figure 2). Since the dinucleotide to be cleaved must be in ssDNA configuration to initiate transfer, we originally hypothesized that MobM could directly recognize and bind the IR2 hairpin (Lorenzo-Díaz et al., 2011). However, a variety of enzymatic and chemical footprinting assays performed on pMV158 scDNA with and without MobM revealed that this was not the case (see below).

### MobM protects *oriT*_pMV158_ from enzymatic digestion and methylation

**Figure 3:**
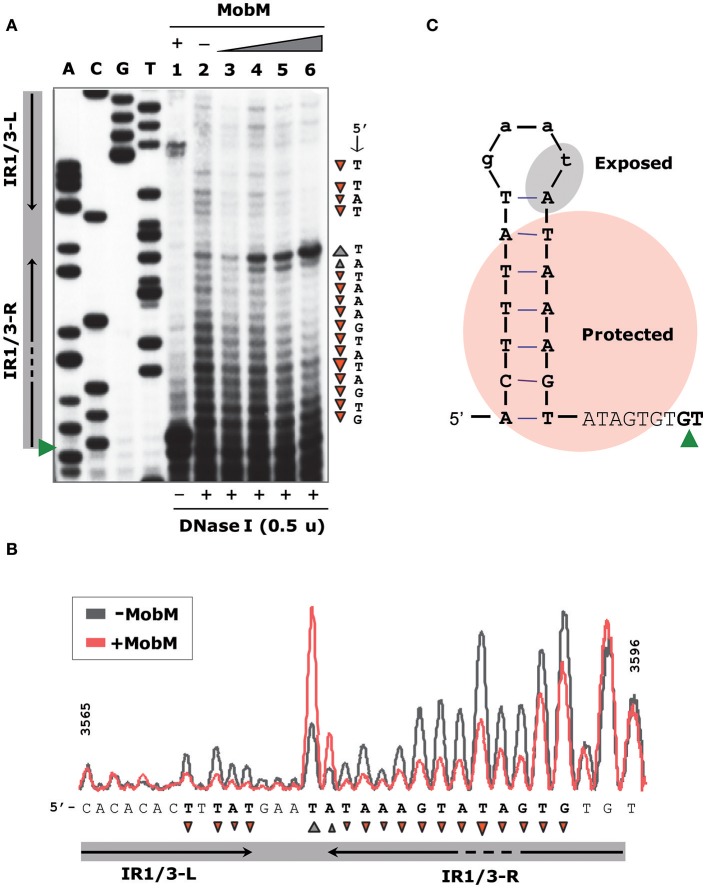
Footprinting of MobM at the *oriT*_pMV158_ assayed by DNase I on pMV158 scDNA. **(A)** Profile of the *oriT*_pMV158_ region (coding strand) digested with DNase I (0.5 units) in the absence and presence of MobM. Binding reactions were treated with DNase I and then subjected to primer extension using the ^32^P-labeled F1 oligonucleotide. Lane 1: control without DNase I to assess the DNA nicking activity of MobM (2 μM) in the DNase I reaction buffer (6 mM MgCl_2_). Lane 2: control without MobM protein, exhibiting the pattern of the naked DNA after DNase I digestion. Lanes 3–6: pMV158 was incubated with increasing concentrations of MobM (0.25, 0.5, 1, and 2 μM, respectively) prior to DNase I digestion. A, C, G, T, Sanger sequencing reactions were prepared using pMV158 and the ^32^P-labeled F1 oligonucleotide. **(B)** Densitometer scans corresponding to lanes 2 (gray line; no MobM) and 6 (red line; 2 μM MobM) are shown. Protected (▾) and hyperexposed (▴) bases to DNase I digestion in the presence of MobM are indicated. **(C)** Schematic representation of the MobM-protected regions on the *oriT*_pMV158_ IR1 structure. Green wedges indicate the position of the 5′-G/T-3′ nick site.

To define the region contacted by MobM at *oriT*_pMV158_ (coding strand), we performed DNase I footprinting assays on pMV158 scDNA, which is its natural target to initiate transfer (Figure 3). MobM-mediated protections were detected by primer extension on the denatured DNase I-digested DNA using the ^32^P-labeled F1 oligonucleotide. This approach resulted in radioactive bands generated by extension of the labeled primer, which is different from the “classical” DNase I digestion of a linear DNA fragment labeled at the 5′-end of one strand. As a consequence, radioactive material does not accumulate at the top of the sequencing gels (Gralla, 1985; Tugores and Brenner, 1994). Two controls were carried out. In the first one, pMV158 scDNA was incubated with MobM but not with DNase I prior to primer extension (Figure 3A, lane 1). In this case, accumulation of a major extension product was observed. Its 3′-end was complementary to the MobM nick site (G3595/T3596; Figure 3B) due to the presence of MgCl_2_ in the reaction buffer. Although Mn^2+^ is the preferred cation of MobM, Mg^2+^ can replace it (Lorenzo-Díaz et al., 2011). The second control was pMV158 scDNA treated with DNase I in the absence of MobM prior to primer extension: cleavage sites along the entire *oriT*_pMV158_ were detected (Figure 3A, lane 2). In the presence of increasing concentrations of MobM, two protected regions spanning IR1-L and IR3-R were observed (Figure 3A, lanes 3–6). Furthermore, at the highest MobM concentrations, DNase I hypersensitive sites were observed in between the left and right arms of IR1/IR3 (corresponding to T3580 and to A3581 positions). These results indicated that either MobM binds to the IR1/IR3 region in double-stranded configuration keeping the internal region accessible to DNase I, or that an IR1/IR3 stem-loop structure is recognized by MobM, so that the stem is protected but the loop is exposed to DNase I cleavage (Figure 3C). Potassium permanganate footprinting assays allowed us to rule out the first hypothesis (below).

**Figure 4:**
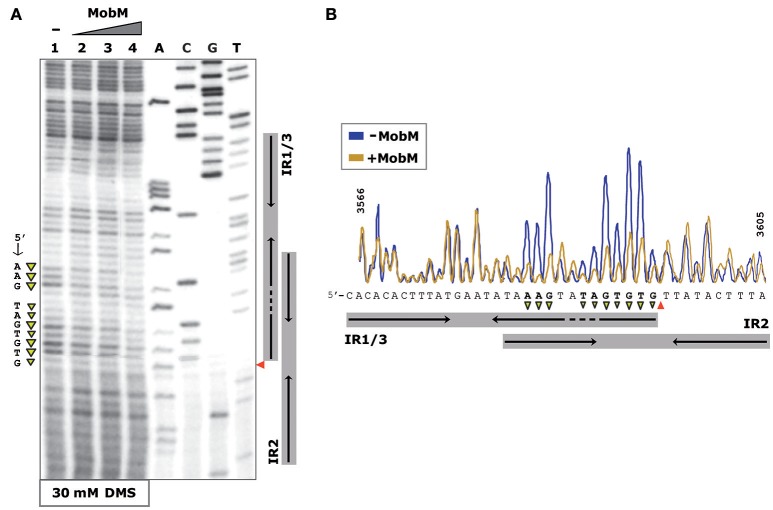
Footprinting of MobM at the *oriT*_pMV158_ (coding strand) assayed by DMS on pMV158 scDNA. **(A)** Profile of the *oriT*_pMV158_ region treated with DMS (30 mM) in the absence and presence of MobM. Lane 1: methylation profile in MobM-untreated samples. Lanes 2–4: pMV158 DNA was incubated with increasing concentrations of MobM (0.5, 1, and 2 μM, respectively). A, C, G, T, Sanger sequencing reactions were prepared using pMV158 and the ^32^P-labeled F1 oligonucleotide. **(B)** Densitometer scans corresponding to lanes 1 (blue line; no MobM), and 4 (yellow line, 2 μM MobM) are shown. The nick site is indicated (red wedges). Protected bases (▾) from DMS methylation in the presence of MobM are indicated.

The interactions between MobM and *oriT*_pMV158_ on pMV158 scDNA were next studied by determination of the bases methylated by DMS in the absence of MobM and in the presence of increasing amounts of MobM (Figure 4). In the presence of MobM, several bases within the right arm of IR3 (mostly A and G) were protected from methylation. Thus, MobM specifically interacts with a site (5′-AAGtaTAGTGTG-3′) located at the right arm of IR3. This site is adjacent to the nick site.

### *oriT*_pMV158_ hairpin dynamics and modulation by relaxase MobM

**Figure 5:**
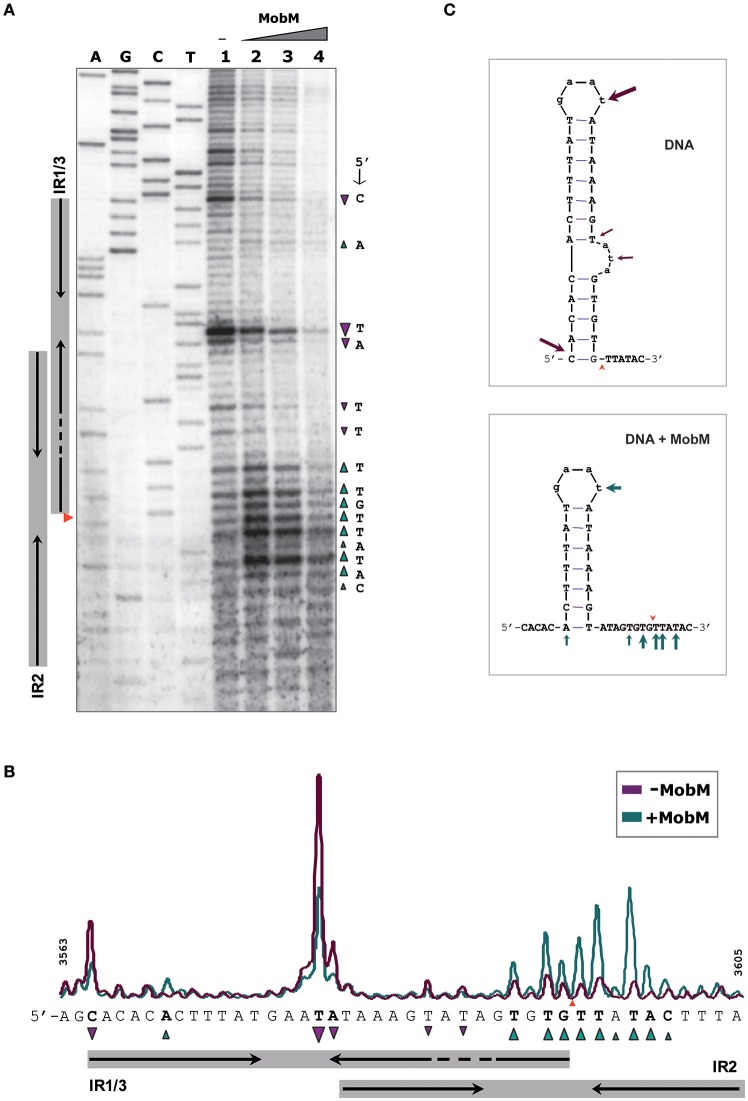
Footprinting of MobM at the *oriT*_pMV158_ (coding strand) assayed by KMnO_4_ on pMV158 scDNA (a footprinting corresponding to the non-coding strand is shown in Supplementary Figure S2). **(A)** Profile of the *oriT*_pMV158_ region (coding strand) treated with KMnO_4_ (5 mM) in the absence and presence of MobM. Lane 1: oxidation profile in samples lacking MobM. Lanes 2–4: pMV158 DNA was incubated with increasing concentrations of MobM (0.5, 1, and 2 μM, respectively). A, C, G, T, Sanger sequencing reactions were prepared using pMV158 and the ^32^P-labeled F1 oligonucleotide; the red wedge to the left points to the 5′-G/T-3′ nick site. **(B)** Densitometer scans corresponding to lanes 1 (purple line; no MobM), and 2 (green line; 0.5 μM MobM). Bases that were hypo- (▾) or hyper-oxidized (▴) by KMnO_4_ are indicated. **(C)** Possible secondary structure of *oriT*_pMV158_ on naked DNA (top panel) and after binding of MobM (bottom panel). Arrows point to the oxidized bases.

To analyse further whether the *oriT*_pMV158_ sequence could undergo particular conformational changes before and after MobM binding, the structure of *oriT*_pMV158_ on scDNA was assessed at nucleotide-level by determination of its reactivity to KMnO_4_ in the absence and presence of MobM. KMnO_4_ reacts with unstacked thymines (and partially with cytosines) in DNA regions such as those forming hairpin-loop structures (Schlax et al., 1995). In the absence of MobM, the thymine located at the loop of IR1/IR3 (T3580) was highly oxidized (Figure 5A, lane 1). Other bases, such as the two thymines located in the bulge loop of IR3 (T3587 and T3589) and the cytosine located at the 5′-end of the hairpin (C3565) were also oxidized. The above results showed that IR3 is extruded as a stem-loop structure (cruciform) on naked scDNA (Figure 5C).

When the experiments were performed in the presence of increasing amounts of MobM, the footprinting profiles changed significantly (Figure 5A, lanes 2-4). In general, it was found that the amount of oxidized bands were “washed out,” this being a common phenomenon when footprints of DNA-binding proteins are assayed at high protein concentrations because of unspecific binding (Tullius and Dombroski, 1986; del Solar et al., 1990). Nevertheless, at the lowest MobM concentration, all the thymines located around the nick site (5′-TGTG/TTATAC-3′) were highly oxidized as compared with the sample that did not receive protein, showing that the binding of MobM led to DNA melting confined to such a region. Further, T3580 was still oxidized in the presence of MobM, as well as the A3570 located at the base of IR1 (Figure 5B). However, the cytosine (C3565) located at the 5′ end of IR3-L (5′-gagCac-3′) showed a significant reduced oxidation compared to the protein-free control sample. Analysis of the structural changes of the non-transferred (non-coding) DNA strand revealed that, upon MobM binding, the specific bases located in the 5′ adjacent region to the complementary IR1 (i.e. the basal stem of IR3 structure) were highly oxidized, whereas the region surrounding the right arm of IR2 kept unchanged (Supplementary Figure S2). Taken together, these results demonstrated that binding of MobM to its cognate *oriT*_pMV158_ switched the IR3 cruciform structure to the hairpin-loop generated by extrusion of IR1 (Figure 5C). Such a MobM-mediated hairpin switch would promote melting of the DNA region adjacent to the nick site, followed by cleavage of the 5′-GpT-3′ dinucleotide and initiation of pMV158 transfer from the donor cell. A summary of the footprinting results is provided in Supplementary Data (Supplementary Figure S3).

### The *oriT*_pMV158_ is widely present in bacteria

The importance of the relaxosome formation in pMV158 goes beyond the plasmid transfer. Embedded within the *oriT*_pMV158_, there are sequences of the two promoters that direct the transcription of *mobM* in different hosts, and that are regulated by MobM (Lorenzo-Díaz et al., 2012; Figure 1B). It was thus interesting to know how outspread were the sequences spanning the *oriT*_pMV158_, and how general their presence was in bacteria. BLASTN searches were done using three regions of the *oriT*_pMV158_ that were characterized previously (Lorenzo-Díaz et al., 2014) and in the present work; these three regions were used as queries in three independent searches, namely the one spanning the entire *oriT*_pMV158_ (“full *oriT*_pMV158_,” 43 nt), the IR3 (31 nt), and the minimal *oriT*_pMV158_, that is the minimal *oriT* sequence needed for MobM binding, which is composed of IR1 plus 8 nt downstream of it (IR1+8, 26 nt) (Lorenzo-Díaz et al., 2011). The search was limited to a maximum number of aligned sequences of 500, and the results are summarized in Table 1. The detailed lineage reports obtained are described in the Supplementary Tables S1–S3. The results showed: (i) a logical agreement between the length of the DNA query and the total number of hits; (ii) the number of hits found in bacteria was similar for the “full *oriT*_pMV158_” and the IR3; (iii) ~50% of such hits were found in *Staphylococcus*, especially in *S. aureus*, where the RCR-plasmids were first reported and characterized (Iordanescu and Surdeanu, 1978; te Riele et al., 1986; Novick, 1989); (iv) the relatively high representation of the *oriT*_pMV158_ in *Enterococcus faecium* (13 hits), and (v) the presence of these sequences in *Enterobacteriales*, including *Escherichia coli*. Whether the amount of hits found in the species of *Staphylococcus* is due to the high number of staphylococcal genomes available is still early to conclude. Be that as it may, the present data mining speaks of a wide distribution of sequences similar to the *oriT*_pMV158_ among bacteria, representing a total of 168 hits for the minimal origin of transfer, IR1+8 (Lorenzo-Díaz et al., 2011). Interestingly, when the query was this latter IR1+8, human sequences with up to ungapped 21 identical nt were retrieved; its significance, if any, is presently unknown (Supplementary Table S3).

**Table 1:** Relevant bacteria represented in the BLAST taxonomy report using the indicated query.

	**Number of hits with the indicated query**		
**Bacteria**	**full *oriT*_pMV158_^a^**	**IR3^b^**	**IR1+8^c^**
**Total hits**	125	119	168
Bacillales	72	70	100
*Staphylococcus aureus*	36	36	37
*Staphylococcus epidermidis*	4	4	4
*Bacillus subtilis*	none	1	7
*Listeria monocytogenes*	none	1	1
Lactobacillales	33	31	43
*Lactobacillus salivarius*	2	2	2
*Enterococcus faecalis*	2	2	2
*Enterococcus faecium*	13	13	13
*Enterococcus cecorum*	5	5	5
*Streptococcus agalactiae*	1	1	5
Enterobacteriales	4	4	4
*Escherichia coli*	3	3	3
*Proteus vulgaris*	1	1	1

The table includes species in which the highest bit score for hits found from that group corresponds to 100% of identity with the query. See Supplementary Tables S1–S3 for more details. Data obtained using the BLASTN 2.7.0+ program (Zhang et al., 2004; Morgulis et al., 2008).

a Query: 5′-GCACACACTTTATGAATATAAAGTATAGTGTGTTATACTTTAC-3′ (43 nt). Total number of hits: bacteria (125), other sequences (14).

b Query: 5′-CACACACTTTATGAATATAAAGTATAGTGTG-3′ (31 nt). Total number of hits: bacteria (119), other sequences (14).

c Query: 5′-ACTTTATGAATATAAAGTATAGTGTG-3-′ (26 nt; minimal oriT_pMV158_; Lorenzo-Díaz et al., 2011). Total number of hits: bacteria (168), eukaryota (316), other sequences (15). Curiously, among the total number of hits with this query, 9 hits were found in Homo sapiens with the highest score of 42.1 (21 identities without gaps).

## Discussion

The relaxase MobM has more than a single role in the biology of pMV158 since it (i) works as the initiator of plasmid transfer (Guzmán and Espinosa, 1997); (ii) participates in the control of plasmid copy number (Lorenzo-Díaz et al., 2017), and (iii) is a transcriptional regulator of its own synthesis (Lorenzo-Díaz et al., 2012). The latter role is not the case for most studied plasmids, which regulate synthesis of their relaxase by accessory proteins (de la Cruz et al., 2010; Ilangovan et al., 2017). The multiple functions of MobM are a sign of the economy of the genetic information characteristic of small plasmids (del Solar et al., 2002), and are facilitated by the compact distribution of regulatory elements within the *oriT*_pMV158_ (Figure 1). MobM seems to recognize its targets in a topology-dependent manner (Lorenzo-Díaz et al., 2011) because: (i) binding of MobM to scDNA or ssDNA is much more efficient than to linear double stranded (ds) DNA; (ii) on ssDNAs, MobM binds to the region encompassing IR1/IR3 with higher affinity (K_d_ ~ 60 nM) than to IR2 (K_d_ >320 nM), and (iii) the minimal *oriT* sequence for MobM binding to ssDNA spans 26 nt containing IR1 plus 8 nt downstream.

Interactions of conjugative relaxases with their cognate *oriT* regions have been studied for several plasmids of Gram-negative hosts. In the case of R100 and R388, both belonging to the family of plasmid F (Cox and Schildbach, 2017), footprintings with KMnO_4_ showed that the nick region melted only upon binding of the relaxase, although the left arm of the single IR (located upstream of the nick site) was not needed for melting (Fukuda and Ohtsubo, 1997; Guasch et al., 2003). A near-atomic resolution cryo-electron microscopy structure of the complex formed by the relaxase TraI_F_ and its cognate *oriT*_F_ has been reported; the structure clarifies the mechanism of transfer in this plasmid, illustrating how the DNA is melted by the relaxase (Ilangovan et al., 2017). In plasmids R1162 and R64, initiation of transfer requires contacts between the relaxase and the inner arm (the closest to the nick site) of the single IR in its cognate *oriT*, but the entire IR is needed for termination (Becker and Meyer, 2000; Furuya and Komano, 2000). Thus, the relaxase of these plasmids appears to recognize dsDNA (inner arm of the IR) and ssDNA (inner and outer arms in hairpin conformation) for initiation and termination of transfer, respectively (Gomis-Rüth and Coll, 2006). However, the situation can be more complex than envisaged because the interaction of some relaxases with their cognate *oriT* can be swapped by three amino acid substitutions (Guja and Schildbach, 2015), and further, a recombination site has been located within the *oriT* of the IncA/C plasmids (Hegyi et al., 2017), these findings indicative of delicate relaxase-*oriT* interactions.

The structure of the *oriT*_pMV158_ is different from the above because it has three IRs (rather than two) and one direct repeat around the nick site (Lorenzo-Díaz et al., 2014). Out of the three IRs, IR2 was predicted to expose the nick site in the loop of a cruciform structure (Figure 2). Since the nick site must be in ssDNA configuration to initiate transfer, we hypothesized that MobM would interact with the IR2-hairpin (Lorenzo-Díaz et al., 2011). However, our present enzymatic and chemical footprintings on pMV158 scDNA rule out such a hypothesis: we have detected the secondary structures formed at *oriT*_pMV158_ in the absence of MobM as well as the MobM-induced conformational changes. In the absence of MobM, IR3 was extruded as a hairpin. Binding of MobM melted the base of IR3 leading to a shift IR3 → IR1 that exposed the dinucleotide 5′-GpT-3′ (nick site) in ssDNA configuration. Regarding IR2, we have not detected formation of an IR2 stem-loop structure on pMV158 scDNA. Moreover, previous analyses performed on ss-oligonucleotides showed that MobM was unable to generate stable complexes with IR2, suggesting that this region might not play a role in the initiation of transfer (Lorenzo-Díaz et al., 2011). However, MobM was able to bind to but not to cleave the IR2 sequence on linear dsDNA (Grohmann et al., 1999). This binding hinders recognition of the *mobM* promoter region by the RNA polymerase, and, therefore, MobM represses the expression of its own gene (Lorenzo-Díaz et al., 2012). *In vivo* studies showed that this self-regulation does not require an intact left arm of IR1/IR3 (Lorenzo-Díaz et al., 2012).

**Figure 6:**
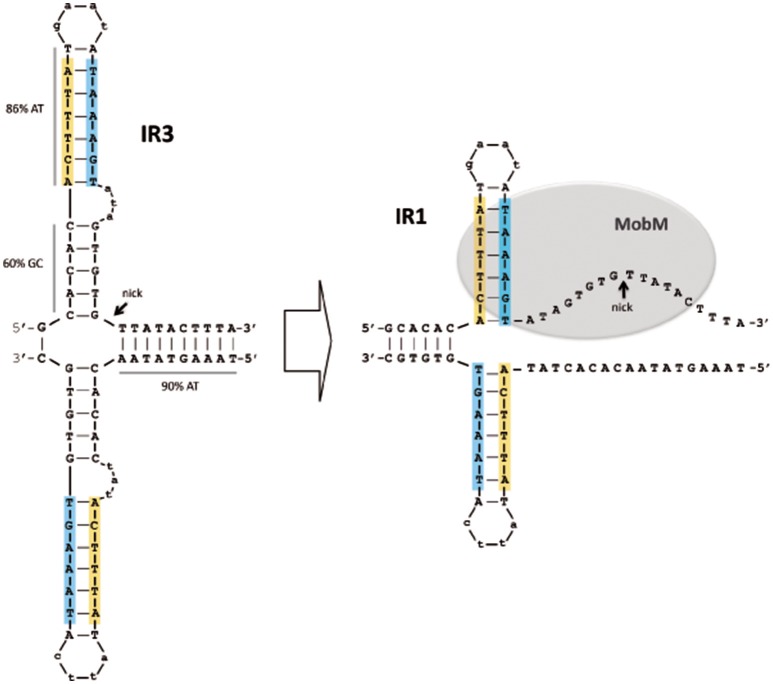
Model of the MobM-mediated hairpin shift at its cognate *oriT*_pMV158_ on scDNA. A cruciform structure containing the IR3 sequence is generated in the naked DNA **(Left)**. After MobM binding, IR3 is displaced to generate IR1, allowing the region around the nick site to be exposed in ssDNA configuration, which would be placed within the MobM active site **(Right)**. In this situation, and assisted by a divalent cation (Mn^2+^) and other amino acid residues, the catalytic residue would be able to cleave the phosphodiester bond of the 5′-GpT-3′ dinucleotide to initiate the plasmid transfer. Potential hairpins generated at the *oriT*_pMV158_ were predicted using the Mfold (3.2) program (http://mfold.rna.albany.edu; Zuker, 2003).

In conclusion, we propose a model that accounts for the dual role of MobM in autoregulation and initiation of transfer of pMV158 and, by extension, to other members of the MGE family (Lorenzo-Díaz et al., 2014). In the donor cell, the three IRs of the *oriT*_pMV158_ could be present either in dsDNA configuration or extruded as a hairpin (Lorenzo-Díaz et al., 2011). Thus, the accessibility of the MobM relaxase to its target would depend on the superhelicity of the plasmid DNA, as it was shown for the RepB replicase of pMV158 (Moscoso et al., 1995). Binding of MobM to IR2 in a dsDNA configuration would preclude extrusion of IR3 and the nick site would be occluded. Thus, expression of the *mobM* gene, as well as transfer of pMV158 would be silenced by means of a single protein-DNA interaction. If this is a steady-state situation, how could MobM promote plasmid transfer? Leaving aside our present ignorance on the signals that trigger conjugation except in a small number of plasmids we propose that, since IR1 is included within IR3 and they partially overlap with IR2, generation of the IR1/IR3 hairpin would impair the binding of MobM to IR2, and *vice versa*. Such a situation would be due to a stochastic distribution of MobM molecules between its target sites so that the relaxase could exploit the conformational variability of hairpin generation (Lilley, 1980; Bikard et al., 2010; Irobalieva et al., 2015). Once IR3 is formed, the binding of MobM would lead to its melting, a likely candidate to start it being the 5′-ATA-3′ internal bulge-loop of IR3, followed by change to the IR1 conformation and concomitant partial melting of the nick region (Figure 6). These changes in hairpin extrusion would allow MobM to cleavage at the nick site, followed by relaxation of plasmid molecules that would become ready to be transferred. Simulation of the three alternative structures at *oriT*_pMV158_ by using oligonucleotides demonstrated that MobM binds preferentially to IR3 and IR1 on single-stranded conformation (Lorenzo-Díaz et al., 2011). Further, the switch of IR3 to IR1 following binding of MobM suggests melting of the region containing the nick sequence and its exposure as ssDNA (Figure 5). Thus, we can conceive the *oriT*_pMV158_ as a molecular switch that would be either turned “*off”* by binding of MobM to IR2 in dsDNA conformation (self-regulation) or turned “*on*” by binding of MobM to the IR3 hairpin and displacement from hairpin IR3 to hairpin IR1, which represents the effective transfer setup.

## Author contributions

All authors participated in the design of the experiments, which were carried out by FL-D, CF-L, and BG-G. ME wrote the first draft, which was corrected by AB and later on by all authors through the successive versions of the manuscript.

### Conflict of interest statement

The authors declare that the research was conducted in the absence of any commercial or financial relationships that could be construed as a potential conflict of interest. The reviewer GdS declared a shared affiliation, with no collaboration, with several of the authors, ME and AB, to the handling Editor.

## Abbreviations

dsDNA: double-stranded DNA
scDNA: supercoiled DNA
ssDNA: single-stranded DNA
HGT: horizontal gene transfer
MGE: mobile genetic elements
RCR: rolling-circle replication
RCR-plasmids: rolling-circle replicating plasmids
nt: nucleotides
IR: inverted repeat.
