# Supplementary material for: Relaxase MobM Induces a Molecular Switch at Its Cognate Origin of Transfer
Source: Front Mol Biosci. 2018 Feb 26;5:17. doi: 10.3389/fmolb.2018.00017 (PMC5863519; doi:10.3389/fmolb.2018.00017)
Supplement: Supplementary file 1 [file Table1.DOC]

**SUPPLEMENTARY DATA**

**Relaxase MobM Induces a Molecular Switch at its Cognate Origin of Transfer**

**Fabián Lorenzo-Díaz, Cris Fernández-López, Beatriz Guillén-Guío, Alicia Bravo*, and Manuel Espinosa***

**Supplementary Figure S1.** The consensus *oriT* sequence derived from the sequences of the 42 members of the pMV158 family of plasmids (group A of the MOB_V1_ family ; Lorenzo-Díaz et al., 2014), was prepared with WebLogo (version 2.8.2; Crooks et al., 2004). Reproduced with permission from Elsevier.


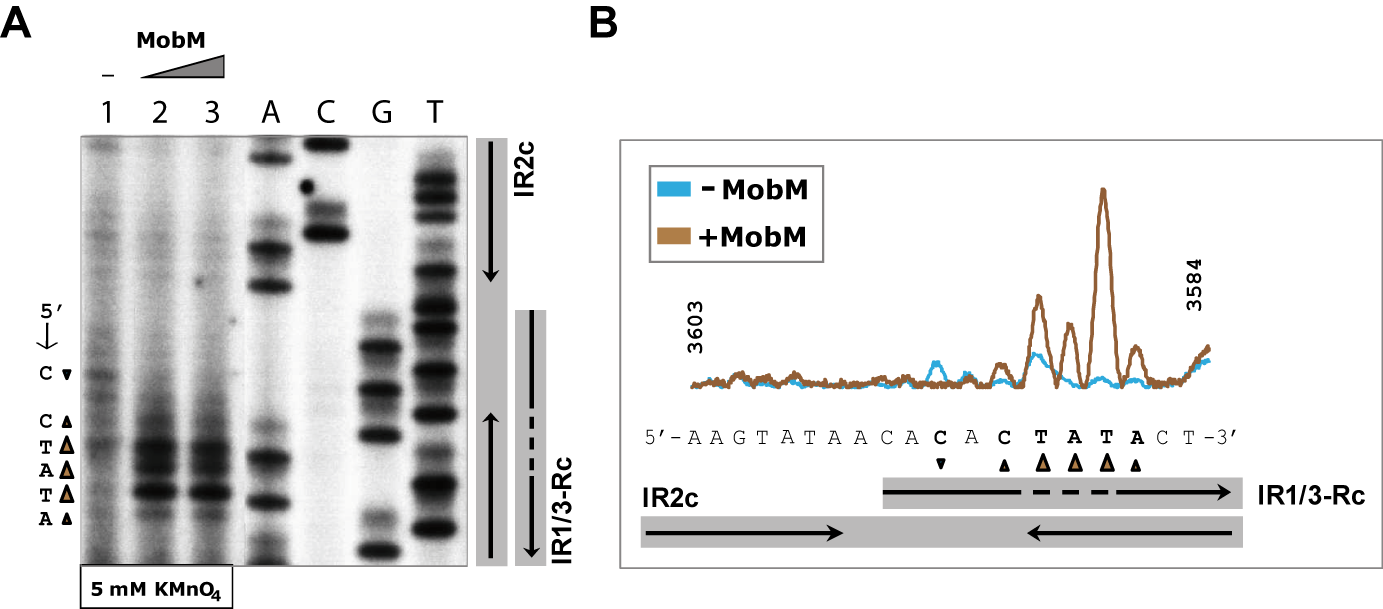


**Supplementary Figure S2.** (A) Profile of the *oriT*_pMV158_ non-coding DNA strand treated with KMnO_4_ (5 mM) in the absence (lane 1) or presence of MobM (1 and 2 µM, lanes 2 and 3, respectively). A, C, G, T: Sanger sequencing reactions were prepared as in Figure 5 but using the ^32^P-labelled PR2 oligonucleotide. (B) Densitometer scans corresponding to lanes 1 (blue line; no MobM), and 3 (brown line; 2 µM of MobM). Bases that were hypo- (▼) or hyper-oxidized (▲) by KMnO_4_ are indicated.

**Supplementary Figure S3:** Summary of the footprints generated by MobM at the *oriT*_pMV158_ on pMV158 scDNA. Direct (orange) and inverted (blue) repeats are highlighted. Bases that were hypo- (▼) or hyper-exposed (▲) are indicated.

**Supplementary Table S1. Lineage report using the ´full *oriT*_pMV158_´ sequence as query** (coordinates 3564 to 3606; GCACACACTTTATGAATATAAAGTATAGTGTGTTATACTTTAC).

| Organism | Blast Name | Score | Number of Hits |
| --- | --- | --- | --- |
| [root](https://www.ncbi.nlm.nih.gov/Taxonomy/Browser/wwwtax.cgi?id=1) |  |  | 139 |
| .[Bacteria](https://www.ncbi.nlm.nih.gov/Taxonomy/Browser/wwwtax.cgi?id=2) | [bacteria](https://www.ncbi.nlm.nih.gov/Taxonomy/Browser/wwwtax.cgi?id=-1) |  | 125 |
| ..[Bacilli](https://www.ncbi.nlm.nih.gov/Taxonomy/Browser/wwwtax.cgi?id=91061) | [firmicutes](https://www.ncbi.nlm.nih.gov/Taxonomy/Browser/wwwtax.cgi?id=1239) |  | 105 |
| ...[Bacillales](https://www.ncbi.nlm.nih.gov/Taxonomy/Browser/wwwtax.cgi?id=1385) | [firmicutes](https://www.ncbi.nlm.nih.gov/Taxonomy/Browser/wwwtax.cgi?id=1239) |  | 72 |
| ....[Staphylococcus](https://www.ncbi.nlm.nih.gov/Taxonomy/Browser/wwwtax.cgi?id=1279) | [firmicutes](https://www.ncbi.nlm.nih.gov/Taxonomy/Browser/wwwtax.cgi?id=1239) |  | 65 |
| .....[Staphylococcus arlettae](https://www.ncbi.nlm.nih.gov/Taxonomy/Browser/wwwtax.cgi?id=29378) | [firmicutes](https://www.ncbi.nlm.nih.gov/Taxonomy/Browser/wwwtax.cgi?id=1239) | 80.5 | 1 |
| .....[Staphylococcus aureus](https://www.ncbi.nlm.nih.gov/Taxonomy/Browser/wwwtax.cgi?id=1280) | [firmicutes](https://www.ncbi.nlm.nih.gov/Taxonomy/Browser/wwwtax.cgi?id=1239) | 80.5 | 36 |
| .....[Staphylococcus sciuri](https://www.ncbi.nlm.nih.gov/Taxonomy/Browser/wwwtax.cgi?id=1296) | [firmicutes](https://www.ncbi.nlm.nih.gov/Taxonomy/Browser/wwwtax.cgi?id=1239) | 80.5 | 1 |
| .....[Staphylococcus capitis](https://www.ncbi.nlm.nih.gov/Taxonomy/Browser/wwwtax.cgi?id=29388) | [firmicutes](https://www.ncbi.nlm.nih.gov/Taxonomy/Browser/wwwtax.cgi?id=1239) | 80.5 | 1 |
| .....[Staphylococcus aureus subsp. aureus](https://www.ncbi.nlm.nih.gov/Taxonomy/Browser/wwwtax.cgi?id=46170) | [firmicutes](https://www.ncbi.nlm.nih.gov/Taxonomy/Browser/wwwtax.cgi?id=1239) | 80.5 | 3 |
| .....[Staphylococcus epidermidis](https://www.ncbi.nlm.nih.gov/Taxonomy/Browser/wwwtax.cgi?id=1282) | [firmicutes](https://www.ncbi.nlm.nih.gov/Taxonomy/Browser/wwwtax.cgi?id=1239) | 80.5 | 4 |
| .....[Staphylococcus aureus subsp. aureus RN4220](https://www.ncbi.nlm.nih.gov/Taxonomy/Browser/wwwtax.cgi?id=561307) | [firmicutes](https://www.ncbi.nlm.nih.gov/Taxonomy/Browser/wwwtax.cgi?id=1239) | 80.5 | 1 |
| .....[Staphylococcus aureus subsp. aureus CN1](https://www.ncbi.nlm.nih.gov/Taxonomy/Browser/wwwtax.cgi?id=1193576) | [firmicutes](https://www.ncbi.nlm.nih.gov/Taxonomy/Browser/wwwtax.cgi?id=1239) | 80.5 | 1 |
| .....[Staphylococcus aureus subsp. aureus ST398](https://www.ncbi.nlm.nih.gov/Taxonomy/Browser/wwwtax.cgi?id=523796) | [firmicutes](https://www.ncbi.nlm.nih.gov/Taxonomy/Browser/wwwtax.cgi?id=1239) | 80.5 | 6 |
| .....[Staphylococcus hyicus](https://www.ncbi.nlm.nih.gov/Taxonomy/Browser/wwwtax.cgi?id=1284) | [firmicutes](https://www.ncbi.nlm.nih.gov/Taxonomy/Browser/wwwtax.cgi?id=1239) | 80.5 | 2 |
| .....[Staphylococcus aureus subsp. aureus ECT-R 2](https://www.ncbi.nlm.nih.gov/Taxonomy/Browser/wwwtax.cgi?id=889933) | [firmicutes](https://www.ncbi.nlm.nih.gov/Taxonomy/Browser/wwwtax.cgi?id=1239) | 80.5 | 1 |
| .....[Staphylococcus aureus 04-02981](https://www.ncbi.nlm.nih.gov/Taxonomy/Browser/wwwtax.cgi?id=703339) | [firmicutes](https://www.ncbi.nlm.nih.gov/Taxonomy/Browser/wwwtax.cgi?id=1239) | 80.5 | 1 |
| .....[Staphylococcus aureus subsp. aureus str. JKD6008](https://www.ncbi.nlm.nih.gov/Taxonomy/Browser/wwwtax.cgi?id=546342) | [firmicutes](https://www.ncbi.nlm.nih.gov/Taxonomy/Browser/wwwtax.cgi?id=1239) | 80.5 | 1 |
| .....[Staphylococcus aureus subsp. aureus Mu3](https://www.ncbi.nlm.nih.gov/Taxonomy/Browser/wwwtax.cgi?id=418127) | [firmicutes](https://www.ncbi.nlm.nih.gov/Taxonomy/Browser/wwwtax.cgi?id=1239) | 80.5 | 1 |
| .....[Staphylococcus aureus subsp. aureus Mu50](https://www.ncbi.nlm.nih.gov/Taxonomy/Browser/wwwtax.cgi?id=158878) | [firmicutes](https://www.ncbi.nlm.nih.gov/Taxonomy/Browser/wwwtax.cgi?id=1239) | 80.5 | 1 |
| .....[Staphylococcus aureus subsp. aureus N315](https://www.ncbi.nlm.nih.gov/Taxonomy/Browser/wwwtax.cgi?id=158879) | [firmicutes](https://www.ncbi.nlm.nih.gov/Taxonomy/Browser/wwwtax.cgi?id=1239) | 80.5 | 1 |
| .....[Staphylococcus cohnii](https://www.ncbi.nlm.nih.gov/Taxonomy/Browser/wwwtax.cgi?id=29382) | [firmicutes](https://www.ncbi.nlm.nih.gov/Taxonomy/Browser/wwwtax.cgi?id=1239) | 80.5 | 1 |
| .....[Staphylococcus schleiferi](https://www.ncbi.nlm.nih.gov/Taxonomy/Browser/wwwtax.cgi?id=1295) | [firmicutes](https://www.ncbi.nlm.nih.gov/Taxonomy/Browser/wwwtax.cgi?id=1239) | 75.0 | 1 |
| .....[Staphylococcus saprophyticus](https://www.ncbi.nlm.nih.gov/Taxonomy/Browser/wwwtax.cgi?id=29385) | [firmicutes](https://www.ncbi.nlm.nih.gov/Taxonomy/Browser/wwwtax.cgi?id=1239) | 75.0 | 1 |
| ....[Ureibacillus thermosphaericus](https://www.ncbi.nlm.nih.gov/Taxonomy/Browser/wwwtax.cgi?id=51173) | [firmicutes](https://www.ncbi.nlm.nih.gov/Taxonomy/Browser/wwwtax.cgi?id=1239) | 80.5 | 1 |
| ....[Bacillus cereus](https://www.ncbi.nlm.nih.gov/Taxonomy/Browser/wwwtax.cgi?id=1396) | [firmicutes](https://www.ncbi.nlm.nih.gov/Taxonomy/Browser/wwwtax.cgi?id=1239) | 80.5 | 1 |
| ....[Bacillus sp.](https://www.ncbi.nlm.nih.gov/Taxonomy/Browser/wwwtax.cgi?id=1409) | [firmicutes](https://www.ncbi.nlm.nih.gov/Taxonomy/Browser/wwwtax.cgi?id=1239) | 80.5 | 2 |
| ....[Bacillus subtilis](https://www.ncbi.nlm.nih.gov/Taxonomy/Browser/wwwtax.cgi?id=1423) | [firmicutes](https://www.ncbi.nlm.nih.gov/Taxonomy/Browser/wwwtax.cgi?id=1239) | 78.7 | 1 |
| ....[Geobacillus stearothermophilus](https://www.ncbi.nlm.nih.gov/Taxonomy/Browser/wwwtax.cgi?id=1422) | [firmicutes](https://www.ncbi.nlm.nih.gov/Taxonomy/Browser/wwwtax.cgi?id=1239) | 75.0 | 1 |
| ....[Listeria monocytogenes](https://www.ncbi.nlm.nih.gov/Taxonomy/Browser/wwwtax.cgi?id=1639) | [firmicutes](https://www.ncbi.nlm.nih.gov/Taxonomy/Browser/wwwtax.cgi?id=1239) | 75.0 | 1 |
| ...[Lactobacillus salivarius](https://www.ncbi.nlm.nih.gov/Taxonomy/Browser/wwwtax.cgi?id=1624) | [firmicutes](https://www.ncbi.nlm.nih.gov/Taxonomy/Browser/wwwtax.cgi?id=1239) | 80.5 | 2 |
| ...[Enterococcus faecalis](https://www.ncbi.nlm.nih.gov/Taxonomy/Browser/wwwtax.cgi?id=1351) | [firmicutes](https://www.ncbi.nlm.nih.gov/Taxonomy/Browser/wwwtax.cgi?id=1239) | 80.5 | 2 |
| ...[Streptococcus pasteurianus](https://www.ncbi.nlm.nih.gov/Taxonomy/Browser/wwwtax.cgi?id=197614) | [firmicutes](https://www.ncbi.nlm.nih.gov/Taxonomy/Browser/wwwtax.cgi?id=1239) | 80.5 | 1 |
| ...[Enterococcus faecium](https://www.ncbi.nlm.nih.gov/Taxonomy/Browser/wwwtax.cgi?id=1352) | [firmicutes](https://www.ncbi.nlm.nih.gov/Taxonomy/Browser/wwwtax.cgi?id=1239) | 80.5 | 13 |
| ...[Enterococcus hirae](https://www.ncbi.nlm.nih.gov/Taxonomy/Browser/wwwtax.cgi?id=1354) | [firmicutes](https://www.ncbi.nlm.nih.gov/Taxonomy/Browser/wwwtax.cgi?id=1239) | 80.5 | 1 |
| ...[Lactobacillus reuteri](https://www.ncbi.nlm.nih.gov/Taxonomy/Browser/wwwtax.cgi?id=1598) | [firmicutes](https://www.ncbi.nlm.nih.gov/Taxonomy/Browser/wwwtax.cgi?id=1239) | 80.5 | 2 |
| ...[Enterococcus cecorum](https://www.ncbi.nlm.nih.gov/Taxonomy/Browser/wwwtax.cgi?id=44008) | [firmicutes](https://www.ncbi.nlm.nih.gov/Taxonomy/Browser/wwwtax.cgi?id=1239) | 80.5 | 5 |
| ...[Enterococcus faecium DO](https://www.ncbi.nlm.nih.gov/Taxonomy/Browser/wwwtax.cgi?id=333849) | [firmicutes](https://www.ncbi.nlm.nih.gov/Taxonomy/Browser/wwwtax.cgi?id=1239) | 80.5 | 1 |
| ...[Streptococcus pasteurianus ATCC 43144](https://www.ncbi.nlm.nih.gov/Taxonomy/Browser/wwwtax.cgi?id=981540) | [firmicutes](https://www.ncbi.nlm.nih.gov/Taxonomy/Browser/wwwtax.cgi?id=1239) | 80.5 | 1 |
| ...[Streptococcus gallolyticus UCN34](https://www.ncbi.nlm.nih.gov/Taxonomy/Browser/wwwtax.cgi?id=637909) | [firmicutes](https://www.ncbi.nlm.nih.gov/Taxonomy/Browser/wwwtax.cgi?id=1239) | 80.5 | 1 |
| ...[Streptococcus suis BM407](https://www.ncbi.nlm.nih.gov/Taxonomy/Browser/wwwtax.cgi?id=568814) | [firmicutes](https://www.ncbi.nlm.nih.gov/Taxonomy/Browser/wwwtax.cgi?id=1239) | 80.5 | 1 |
| ...[Streptococcus agalactiae](https://www.ncbi.nlm.nih.gov/Taxonomy/Browser/wwwtax.cgi?id=1311) | [firmicutes](https://www.ncbi.nlm.nih.gov/Taxonomy/Browser/wwwtax.cgi?id=1239) | 80.5 | 1 |
| ...[Lactobacillus johnsonii](https://www.ncbi.nlm.nih.gov/Taxonomy/Browser/wwwtax.cgi?id=33959) | [firmicutes](https://www.ncbi.nlm.nih.gov/Taxonomy/Browser/wwwtax.cgi?id=1239) | 75.0 | 1 |
| ...[Streptococcus suis D12](https://www.ncbi.nlm.nih.gov/Taxonomy/Browser/wwwtax.cgi?id=1004952) | [firmicutes](https://www.ncbi.nlm.nih.gov/Taxonomy/Browser/wwwtax.cgi?id=1239) | 52.8 | 1 |
| ..[uncultured bacterium](https://www.ncbi.nlm.nih.gov/Taxonomy/Browser/wwwtax.cgi?id=77133) | [bacteria](https://www.ncbi.nlm.nih.gov/Taxonomy/Browser/wwwtax.cgi?id=-1) | 80.5 | 15 |
| ..[Escherichia coli](https://www.ncbi.nlm.nih.gov/Taxonomy/Browser/wwwtax.cgi?id=562) | [enterobacteria](https://www.ncbi.nlm.nih.gov/Taxonomy/Browser/wwwtax.cgi?id=91347) | 80.5 | 3 |
| ..[Proteus vulgaris](https://www.ncbi.nlm.nih.gov/Taxonomy/Browser/wwwtax.cgi?id=585) | [enterobacteria](https://www.ncbi.nlm.nih.gov/Taxonomy/Browser/wwwtax.cgi?id=91347) | 80.5 | 1 |
| ..[uncultured bacterium MID12](https://www.ncbi.nlm.nih.gov/Taxonomy/Browser/wwwtax.cgi?id=764346) | [bacteria](https://www.ncbi.nlm.nih.gov/Taxonomy/Browser/wwwtax.cgi?id=-1) | 80.5 | 1 |
| .[Cloning vector pME10](https://www.ncbi.nlm.nih.gov/Taxonomy/Browser/wwwtax.cgi?id=1770536) | [other sequences](https://www.ncbi.nlm.nih.gov/Taxonomy/Browser/wwwtax.cgi?id=28384) | 80.5 | 1 |
| .[S. aureus-E. coli shuttle vector pBUS1-Pcap-HC](https://www.ncbi.nlm.nih.gov/Taxonomy/Browser/wwwtax.cgi?id=1545807) | [other sequences](https://www.ncbi.nlm.nih.gov/Taxonomy/Browser/wwwtax.cgi?id=28384) | 80.5 | 1 |
| .[Expression vector pME9](https://www.ncbi.nlm.nih.gov/Taxonomy/Browser/wwwtax.cgi?id=1296589) | [other sequences](https://www.ncbi.nlm.nih.gov/Taxonomy/Browser/wwwtax.cgi?id=28384) | 80.5 | 1 |
| .[Shuttle vector pME8](https://www.ncbi.nlm.nih.gov/Taxonomy/Browser/wwwtax.cgi?id=1296590) | [other sequences](https://www.ncbi.nlm.nih.gov/Taxonomy/Browser/wwwtax.cgi?id=28384) | 80.5 | 1 |
| .[Expression vector pME10](https://www.ncbi.nlm.nih.gov/Taxonomy/Browser/wwwtax.cgi?id=1296588) | [other sequences](https://www.ncbi.nlm.nih.gov/Taxonomy/Browser/wwwtax.cgi?id=28384) | 80.5 | 1 |
| .[GFP expression vector pHapII](https://www.ncbi.nlm.nih.gov/Taxonomy/Browser/wwwtax.cgi?id=866769) | [other sequences](https://www.ncbi.nlm.nih.gov/Taxonomy/Browser/wwwtax.cgi?id=28384) | 80.5 | 1 |
| .[Shuttle vector pMK3](https://www.ncbi.nlm.nih.gov/Taxonomy/Browser/wwwtax.cgi?id=515189) | [other sequences](https://www.ncbi.nlm.nih.gov/Taxonomy/Browser/wwwtax.cgi?id=28384) | 80.5 | 1 |
| .[Shuttle expression-secretion vector pP43NMK](https://www.ncbi.nlm.nih.gov/Taxonomy/Browser/wwwtax.cgi?id=370428) | [other sequences](https://www.ncbi.nlm.nih.gov/Taxonomy/Browser/wwwtax.cgi?id=28384) | 80.5 | 1 |
| .[Cloning vector pND50](https://www.ncbi.nlm.nih.gov/Taxonomy/Browser/wwwtax.cgi?id=316466) | [other sequences](https://www.ncbi.nlm.nih.gov/Taxonomy/Browser/wwwtax.cgi?id=28384) | 80.5 | 1 |
| .[Shuttle vector pHY320PLK](https://www.ncbi.nlm.nih.gov/Taxonomy/Browser/wwwtax.cgi?id=77677) | [other sequences](https://www.ncbi.nlm.nih.gov/Taxonomy/Browser/wwwtax.cgi?id=28384) | 80.5 | 1 |
| .[Plasmid pUB110](https://www.ncbi.nlm.nih.gov/Taxonomy/Browser/wwwtax.cgi?id=2655) | [other sequences](https://www.ncbi.nlm.nih.gov/Taxonomy/Browser/wwwtax.cgi?id=28384) | 80.5 | 2 |
| **.**[**Plasmid pMV158**](https://www.ncbi.nlm.nih.gov/Taxonomy/Browser/wwwtax.cgi?id=2606) | [**other sequences**](https://www.ncbi.nlm.nih.gov/Taxonomy/Browser/wwwtax.cgi?id=28384) | **80.5** | **1** |
| .[synthetic construct](https://www.ncbi.nlm.nih.gov/Taxonomy/Browser/wwwtax.cgi?id=32630) | [other sequences](https://www.ncbi.nlm.nih.gov/Taxonomy/Browser/wwwtax.cgi?id=28384) | 78.7 | 1 |

Program: BLASTN 2.7.0+ (Zhang et al., 2004; Morgulis et al., 2008). Search database Nucleotide collection (nr/nt) using Megablast, on October 1^st^, 2017. Maximum number of aligned sequences to display: 500. Score 80.5 (100% identity).

**Supplementary Table S2. Lineage report using the IR3 sequence as query** (coordinates 3565 to 3595; CACACACTTTATGAATATAAAGTATAGTGTG).

| Organism | Blast Name | Score | Number of Hits |
| --- | --- | --- | --- |
| [root](https://www.ncbi.nlm.nih.gov/Taxonomy/Browser/wwwtax.cgi?id=1) |  |  | 133 |
| **.**[Bacteria](https://www.ncbi.nlm.nih.gov/Taxonomy/Browser/wwwtax.cgi?id=2) | [bacteria](https://www.ncbi.nlm.nih.gov/Taxonomy/Browser/wwwtax.cgi?id=-1) |  | 119 |
| **..**[Bacilli](https://www.ncbi.nlm.nih.gov/Taxonomy/Browser/wwwtax.cgi?id=91061) | [firmicutes](https://www.ncbi.nlm.nih.gov/Taxonomy/Browser/wwwtax.cgi?id=1239) |  | 101 |
| **...**[Bacillales](https://www.ncbi.nlm.nih.gov/Taxonomy/Browser/wwwtax.cgi?id=1385) | [firmicutes](https://www.ncbi.nlm.nih.gov/Taxonomy/Browser/wwwtax.cgi?id=1239) |  | 70 |
| **....**[Staphylococcus](https://www.ncbi.nlm.nih.gov/Taxonomy/Browser/wwwtax.cgi?id=1279) | [firmicutes](https://www.ncbi.nlm.nih.gov/Taxonomy/Browser/wwwtax.cgi?id=1239) |  | 63 |
| **.....**[Staphylococcus arlettae](https://www.ncbi.nlm.nih.gov/Taxonomy/Browser/wwwtax.cgi?id=29378) | [firmicutes](https://www.ncbi.nlm.nih.gov/Taxonomy/Browser/wwwtax.cgi?id=1239) | 58.4 | 1 |
| **.....**[Staphylococcus aureus](https://www.ncbi.nlm.nih.gov/Taxonomy/Browser/wwwtax.cgi?id=1280) | [firmicutes](https://www.ncbi.nlm.nih.gov/Taxonomy/Browser/wwwtax.cgi?id=1239) | 58.4 | 36 |
| **.....**[Staphylococcus epidermidis](https://www.ncbi.nlm.nih.gov/Taxonomy/Browser/wwwtax.cgi?id=1282) | [firmicutes](https://www.ncbi.nlm.nih.gov/Taxonomy/Browser/wwwtax.cgi?id=1239) | 58.4 | 4 |
| **.....**[Staphylococcus sciuri](https://www.ncbi.nlm.nih.gov/Taxonomy/Browser/wwwtax.cgi?id=1296) | [firmicutes](https://www.ncbi.nlm.nih.gov/Taxonomy/Browser/wwwtax.cgi?id=1239) | 58.4 | 1 |
| **.....**[Staphylococcus capitis](https://www.ncbi.nlm.nih.gov/Taxonomy/Browser/wwwtax.cgi?id=29388) | [firmicutes](https://www.ncbi.nlm.nih.gov/Taxonomy/Browser/wwwtax.cgi?id=1239) | 58.4 | 1 |
| **.....**[Staphylococcus aureus subsp. aureus](https://www.ncbi.nlm.nih.gov/Taxonomy/Browser/wwwtax.cgi?id=46170) | [firmicutes](https://www.ncbi.nlm.nih.gov/Taxonomy/Browser/wwwtax.cgi?id=1239) | 58.4 | 3 |
| **.....**[Staphylococcus aureus subsp. aureus RN4220](https://www.ncbi.nlm.nih.gov/Taxonomy/Browser/wwwtax.cgi?id=561307) | [firmicutes](https://www.ncbi.nlm.nih.gov/Taxonomy/Browser/wwwtax.cgi?id=1239) | 58.4 | 1 |
| **.....**[Staphylococcus aureus subsp. aureus CN1](https://www.ncbi.nlm.nih.gov/Taxonomy/Browser/wwwtax.cgi?id=1193576) | [firmicutes](https://www.ncbi.nlm.nih.gov/Taxonomy/Browser/wwwtax.cgi?id=1239) | 58.4 | 1 |
| **.....**[Staphylococcus aureus subsp. aureus ST398](https://www.ncbi.nlm.nih.gov/Taxonomy/Browser/wwwtax.cgi?id=523796) | [firmicutes](https://www.ncbi.nlm.nih.gov/Taxonomy/Browser/wwwtax.cgi?id=1239) | 58.4 | 6 |
| **.....**[Staphylococcus hyicus](https://www.ncbi.nlm.nih.gov/Taxonomy/Browser/wwwtax.cgi?id=1284) | [firmicutes](https://www.ncbi.nlm.nih.gov/Taxonomy/Browser/wwwtax.cgi?id=1239) | 58.4 | 1 |
| **.....**[Staphylococcus aureus subsp. aureus ECT-R 2](https://www.ncbi.nlm.nih.gov/Taxonomy/Browser/wwwtax.cgi?id=889933) | [firmicutes](https://www.ncbi.nlm.nih.gov/Taxonomy/Browser/wwwtax.cgi?id=1239) | 58.4 | 1 |
| **.....**[Staphylococcus aureus 04-02981](https://www.ncbi.nlm.nih.gov/Taxonomy/Browser/wwwtax.cgi?id=703339) | [firmicutes](https://www.ncbi.nlm.nih.gov/Taxonomy/Browser/wwwtax.cgi?id=1239) | 58.4 | 1 |
| **.....**[Staphylococcus aureus subsp. aureus str. JKD6008](https://www.ncbi.nlm.nih.gov/Taxonomy/Browser/wwwtax.cgi?id=546342) | [firmicutes](https://www.ncbi.nlm.nih.gov/Taxonomy/Browser/wwwtax.cgi?id=1239) | 58.4 | 1 |
| **.....**[Staphylococcus aureus subsp. aureus Mu3](https://www.ncbi.nlm.nih.gov/Taxonomy/Browser/wwwtax.cgi?id=418127) | [firmicutes](https://www.ncbi.nlm.nih.gov/Taxonomy/Browser/wwwtax.cgi?id=1239) | 58.4 | 1 |
| **.....**[Staphylococcus aureus subsp. aureus Mu50](https://www.ncbi.nlm.nih.gov/Taxonomy/Browser/wwwtax.cgi?id=158878) | [firmicutes](https://www.ncbi.nlm.nih.gov/Taxonomy/Browser/wwwtax.cgi?id=1239) | 58.4 | 1 |
| **.....**[Staphylococcus aureus subsp. aureus N315](https://www.ncbi.nlm.nih.gov/Taxonomy/Browser/wwwtax.cgi?id=158879) | [firmicutes](https://www.ncbi.nlm.nih.gov/Taxonomy/Browser/wwwtax.cgi?id=1239) | 58.4 | 1 |
| **.....**[Staphylococcus cohnii](https://www.ncbi.nlm.nih.gov/Taxonomy/Browser/wwwtax.cgi?id=29382) | [firmicutes](https://www.ncbi.nlm.nih.gov/Taxonomy/Browser/wwwtax.cgi?id=1239) | 58.4 | 1 |
| **.....**[Staphylococcus schleiferi](https://www.ncbi.nlm.nih.gov/Taxonomy/Browser/wwwtax.cgi?id=1295) | [firmicutes](https://www.ncbi.nlm.nih.gov/Taxonomy/Browser/wwwtax.cgi?id=1239) | 52.8 | 1 |
| **....**[Ureibacillus thermosphaericus](https://www.ncbi.nlm.nih.gov/Taxonomy/Browser/wwwtax.cgi?id=51173) | [firmicutes](https://www.ncbi.nlm.nih.gov/Taxonomy/Browser/wwwtax.cgi?id=1239) | 58.4 | 1 |
| **....**[Geobacillus stearothermophilus](https://www.ncbi.nlm.nih.gov/Taxonomy/Browser/wwwtax.cgi?id=1422) | [firmicutes](https://www.ncbi.nlm.nih.gov/Taxonomy/Browser/wwwtax.cgi?id=1239) | 58.4 | 1 |
| **....**[Bacillus sp.](https://www.ncbi.nlm.nih.gov/Taxonomy/Browser/wwwtax.cgi?id=1409) | [firmicutes](https://www.ncbi.nlm.nih.gov/Taxonomy/Browser/wwwtax.cgi?id=1239) | 58.4 | 2 |
| **....**[Bacillus subtilis](https://www.ncbi.nlm.nih.gov/Taxonomy/Browser/wwwtax.cgi?id=1423) | [firmicutes](https://www.ncbi.nlm.nih.gov/Taxonomy/Browser/wwwtax.cgi?id=1239) | 58.4 | 1 |
| **....**[Listeria monocytogenes](https://www.ncbi.nlm.nih.gov/Taxonomy/Browser/wwwtax.cgi?id=1639) | [firmicutes](https://www.ncbi.nlm.nih.gov/Taxonomy/Browser/wwwtax.cgi?id=1239) | 58.4 | 1 |
| **....**[Bacillus cereus](https://www.ncbi.nlm.nih.gov/Taxonomy/Browser/wwwtax.cgi?id=1396) | [firmicutes](https://www.ncbi.nlm.nih.gov/Taxonomy/Browser/wwwtax.cgi?id=1239) | 58.4 | 1 |
| **...**[Lactobacillus salivarius](https://www.ncbi.nlm.nih.gov/Taxonomy/Browser/wwwtax.cgi?id=1624) | [firmicutes](https://www.ncbi.nlm.nih.gov/Taxonomy/Browser/wwwtax.cgi?id=1239) | 58.4 | 2 |
| **...**[Enterococcus faecalis](https://www.ncbi.nlm.nih.gov/Taxonomy/Browser/wwwtax.cgi?id=1351) | [firmicutes](https://www.ncbi.nlm.nih.gov/Taxonomy/Browser/wwwtax.cgi?id=1239) | 58.4 | 2 |
| **...**[Streptococcus pasteurianus](https://www.ncbi.nlm.nih.gov/Taxonomy/Browser/wwwtax.cgi?id=197614) | [firmicutes](https://www.ncbi.nlm.nih.gov/Taxonomy/Browser/wwwtax.cgi?id=1239) | 58.4 | 1 |
| **...**[Enterococcus faecium](https://www.ncbi.nlm.nih.gov/Taxonomy/Browser/wwwtax.cgi?id=1352) | [firmicutes](https://www.ncbi.nlm.nih.gov/Taxonomy/Browser/wwwtax.cgi?id=1239) | 58.4 | 13 |
| **...**[Lactobacillus johnsonii](https://www.ncbi.nlm.nih.gov/Taxonomy/Browser/wwwtax.cgi?id=33959) | [firmicutes](https://www.ncbi.nlm.nih.gov/Taxonomy/Browser/wwwtax.cgi?id=1239) | 58.4 | 1 |
| **...**[Enterococcus hirae](https://www.ncbi.nlm.nih.gov/Taxonomy/Browser/wwwtax.cgi?id=1354) | [firmicutes](https://www.ncbi.nlm.nih.gov/Taxonomy/Browser/wwwtax.cgi?id=1239) | 58.4 | 1 |
| **...**[Lactobacillus reuteri](https://www.ncbi.nlm.nih.gov/Taxonomy/Browser/wwwtax.cgi?id=1598) | [firmicutes](https://www.ncbi.nlm.nih.gov/Taxonomy/Browser/wwwtax.cgi?id=1239) | 58.4 | 1 |
| **...**[Enterococcus cecorum](https://www.ncbi.nlm.nih.gov/Taxonomy/Browser/wwwtax.cgi?id=44008) | [firmicutes](https://www.ncbi.nlm.nih.gov/Taxonomy/Browser/wwwtax.cgi?id=1239) | 58.4 | 5 |
| **...**[Enterococcus faecium DO](https://www.ncbi.nlm.nih.gov/Taxonomy/Browser/wwwtax.cgi?id=333849) | [firmicutes](https://www.ncbi.nlm.nih.gov/Taxonomy/Browser/wwwtax.cgi?id=1239) | 58.4 | 1 |
| **...**[Streptococcus pasteurianus ATCC 43144](https://www.ncbi.nlm.nih.gov/Taxonomy/Browser/wwwtax.cgi?id=981540) | [firmicutes](https://www.ncbi.nlm.nih.gov/Taxonomy/Browser/wwwtax.cgi?id=1239) | 58.4 | 1 |
| **...**[Streptococcus gallolyticus UCN34](https://www.ncbi.nlm.nih.gov/Taxonomy/Browser/wwwtax.cgi?id=637909) | [firmicutes](https://www.ncbi.nlm.nih.gov/Taxonomy/Browser/wwwtax.cgi?id=1239) | 58.4 | 1 |
| **...**[Streptococcus suis BM407](https://www.ncbi.nlm.nih.gov/Taxonomy/Browser/wwwtax.cgi?id=568814) | [firmicutes](https://www.ncbi.nlm.nih.gov/Taxonomy/Browser/wwwtax.cgi?id=1239) | 58.4 | 1 |
| **...**[Streptococcus agalactiae](https://www.ncbi.nlm.nih.gov/Taxonomy/Browser/wwwtax.cgi?id=1311) | [firmicutes](https://www.ncbi.nlm.nih.gov/Taxonomy/Browser/wwwtax.cgi?id=1239) | 58.4 | 1 |
| **..**[uncultured bacterium](https://www.ncbi.nlm.nih.gov/Taxonomy/Browser/wwwtax.cgi?id=77133) | [bacteria](https://www.ncbi.nlm.nih.gov/Taxonomy/Browser/wwwtax.cgi?id=-1) | 58.4 | 13 |
| **..**[Escherichia coli](https://www.ncbi.nlm.nih.gov/Taxonomy/Browser/wwwtax.cgi?id=562) | [enterobacteria](https://www.ncbi.nlm.nih.gov/Taxonomy/Browser/wwwtax.cgi?id=91347) | 58.4 | 3 |
| **..**[Proteus vulgaris](https://www.ncbi.nlm.nih.gov/Taxonomy/Browser/wwwtax.cgi?id=585) | [enterobacteria](https://www.ncbi.nlm.nih.gov/Taxonomy/Browser/wwwtax.cgi?id=91347) | 58.4 | 1 |
| **..**[uncultured bacterium MID12](https://www.ncbi.nlm.nih.gov/Taxonomy/Browser/wwwtax.cgi?id=764346) | [bacteria](https://www.ncbi.nlm.nih.gov/Taxonomy/Browser/wwwtax.cgi?id=-1) | 58.4 | 1 |
| **.**[Cloning vector pME10](https://www.ncbi.nlm.nih.gov/Taxonomy/Browser/wwwtax.cgi?id=1770536) | [other sequences](https://www.ncbi.nlm.nih.gov/Taxonomy/Browser/wwwtax.cgi?id=28384) | 58.4 | 1 |
| **.**[S. aureus-E. coli shuttle vector pBUS1-Pcap-HC](https://www.ncbi.nlm.nih.gov/Taxonomy/Browser/wwwtax.cgi?id=1545807) | [other sequences](https://www.ncbi.nlm.nih.gov/Taxonomy/Browser/wwwtax.cgi?id=28384) | 58.4 | 1 |
| **.**[Expression vector pME9](https://www.ncbi.nlm.nih.gov/Taxonomy/Browser/wwwtax.cgi?id=1296589) | [other sequences](https://www.ncbi.nlm.nih.gov/Taxonomy/Browser/wwwtax.cgi?id=28384) | 58.4 | 1 |
| **.**[Shuttle vector pME8](https://www.ncbi.nlm.nih.gov/Taxonomy/Browser/wwwtax.cgi?id=1296590) | [other sequences](https://www.ncbi.nlm.nih.gov/Taxonomy/Browser/wwwtax.cgi?id=28384) | 58.4 | 1 |
| **.**[Expression vector pME10](https://www.ncbi.nlm.nih.gov/Taxonomy/Browser/wwwtax.cgi?id=1296588) | [other sequences](https://www.ncbi.nlm.nih.gov/Taxonomy/Browser/wwwtax.cgi?id=28384) | 58.4 | 1 |
| **.**[GFP expression vector pHapII](https://www.ncbi.nlm.nih.gov/Taxonomy/Browser/wwwtax.cgi?id=866769) | [other sequences](https://www.ncbi.nlm.nih.gov/Taxonomy/Browser/wwwtax.cgi?id=28384) | 58.4 | 1 |
| **.**[Shuttle vector pMK3](https://www.ncbi.nlm.nih.gov/Taxonomy/Browser/wwwtax.cgi?id=515189) | [other sequences](https://www.ncbi.nlm.nih.gov/Taxonomy/Browser/wwwtax.cgi?id=28384) | 58.4 | 1 |
| **.**[Shuttle expression-secretion vector pP43NMK](https://www.ncbi.nlm.nih.gov/Taxonomy/Browser/wwwtax.cgi?id=370428) | [other sequences](https://www.ncbi.nlm.nih.gov/Taxonomy/Browser/wwwtax.cgi?id=28384) | 58.4 | 1 |
| **.**[Cloning vector pND50](https://www.ncbi.nlm.nih.gov/Taxonomy/Browser/wwwtax.cgi?id=316466) | [other sequences](https://www.ncbi.nlm.nih.gov/Taxonomy/Browser/wwwtax.cgi?id=28384) | 58.4 | 1 |
| **.**[synthetic construct](https://www.ncbi.nlm.nih.gov/Taxonomy/Browser/wwwtax.cgi?id=32630) | [other sequences](https://www.ncbi.nlm.nih.gov/Taxonomy/Browser/wwwtax.cgi?id=28384) | 58.4 | 1 |
| **.**[Shuttle vector pHY320PLK](https://www.ncbi.nlm.nih.gov/Taxonomy/Browser/wwwtax.cgi?id=77677) | [other sequences](https://www.ncbi.nlm.nih.gov/Taxonomy/Browser/wwwtax.cgi?id=28384) | 58.4 | 1 |
| **.**[Plasmid pUB110](https://www.ncbi.nlm.nih.gov/Taxonomy/Browser/wwwtax.cgi?id=2655) | [other sequences](https://www.ncbi.nlm.nih.gov/Taxonomy/Browser/wwwtax.cgi?id=28384) | 58.4 | 2 |
| **.**[**Plasmid pMV158**](https://www.ncbi.nlm.nih.gov/Taxonomy/Browser/wwwtax.cgi?id=2606) | [**other sequences**](https://www.ncbi.nlm.nih.gov/Taxonomy/Browser/wwwtax.cgi?id=28384) | **58.4** | **1** |

Program: BLASTN 2.7.0+ (Zhang et al., 2004; Morgulis et al., 2008). Search database Nucleotide collection (nr/nt) using Megablast, on October 1^st^, 2017. Maximum number of aligned sequences to display: 500. Score 58.4 (100% identity).

**Supplementary Table S3. Lineage report using the IR1+8 sequence as query** (coordinates 3570 to 3595; ACTTTATGAATATAAAGTATAGTGTG).

| Organism | Blast Name | Score | Number of Hits |
| --- | --- | --- | --- |
| [root](https://www.ncbi.nlm.nih.gov/Taxonomy/Browser/wwwtax.cgi?id=1) |  |  | 504 |
| .[cellular organisms](https://www.ncbi.nlm.nih.gov/Taxonomy/Browser/wwwtax.cgi?id=131567) |  |  | 487 |
| ..[Bacteria](https://www.ncbi.nlm.nih.gov/Taxonomy/Browser/wwwtax.cgi?id=2) | [bacteria](https://www.ncbi.nlm.nih.gov/Taxonomy/Browser/wwwtax.cgi?id=-1) |  | 168 |
| ...[Firmicutes](https://www.ncbi.nlm.nih.gov/Taxonomy/Browser/wwwtax.cgi?id=1239) | [firmicutes](https://www.ncbi.nlm.nih.gov/Taxonomy/Browser/wwwtax.cgi?id=1239) |  | 144 |
| ....[Bacilli](https://www.ncbi.nlm.nih.gov/Taxonomy/Browser/wwwtax.cgi?id=91061) | [firmicutes](https://www.ncbi.nlm.nih.gov/Taxonomy/Browser/wwwtax.cgi?id=1239) |  | 143 |
| .....[Bacillales](https://www.ncbi.nlm.nih.gov/Taxonomy/Browser/wwwtax.cgi?id=1385) | [firmicutes](https://www.ncbi.nlm.nih.gov/Taxonomy/Browser/wwwtax.cgi?id=1239) |  | 100 |
| ......[Staphylococcus](https://www.ncbi.nlm.nih.gov/Taxonomy/Browser/wwwtax.cgi?id=1279) | [firmicutes](https://www.ncbi.nlm.nih.gov/Taxonomy/Browser/wwwtax.cgi?id=1239) |  | 81 |
| .......[Staphylococcus arlettae](https://www.ncbi.nlm.nih.gov/Taxonomy/Browser/wwwtax.cgi?id=29378) | [firmicutes](https://www.ncbi.nlm.nih.gov/Taxonomy/Browser/wwwtax.cgi?id=1239) | 52.0 | 2 |
| .......[Staphylococcus aureus](https://www.ncbi.nlm.nih.gov/Taxonomy/Browser/wwwtax.cgi?id=1280) | [firmicutes](https://www.ncbi.nlm.nih.gov/Taxonomy/Browser/wwwtax.cgi?id=1239) | 52.0 | 37 |
| .......[Staphylococcus epidermidis](https://www.ncbi.nlm.nih.gov/Taxonomy/Browser/wwwtax.cgi?id=1282) | [firmicutes](https://www.ncbi.nlm.nih.gov/Taxonomy/Browser/wwwtax.cgi?id=1239) | 52.0 | 4 |
| .......[Staphylococcus sciuri](https://www.ncbi.nlm.nih.gov/Taxonomy/Browser/wwwtax.cgi?id=1296) | [firmicutes](https://www.ncbi.nlm.nih.gov/Taxonomy/Browser/wwwtax.cgi?id=1239) | 52.0 | 2 |
| .......[Staphylococcus capitis](https://www.ncbi.nlm.nih.gov/Taxonomy/Browser/wwwtax.cgi?id=29388) | [firmicutes](https://www.ncbi.nlm.nih.gov/Taxonomy/Browser/wwwtax.cgi?id=1239) | 52.0 | 1 |
| .......[Staphylococcus aureus subsp. aureus](https://www.ncbi.nlm.nih.gov/Taxonomy/Browser/wwwtax.cgi?id=46170) | [firmicutes](https://www.ncbi.nlm.nih.gov/Taxonomy/Browser/wwwtax.cgi?id=1239) | 52.0 | 13 |
| .......[Staphylococcus aureus subsp. aureus RN4220](https://www.ncbi.nlm.nih.gov/Taxonomy/Browser/wwwtax.cgi?id=561307) | [firmicutes](https://www.ncbi.nlm.nih.gov/Taxonomy/Browser/wwwtax.cgi?id=1239) | 52.0 | 1 |
| .......[Staphylococcus aureus subsp. aureus CN1](https://www.ncbi.nlm.nih.gov/Taxonomy/Browser/wwwtax.cgi?id=1193576) | [firmicutes](https://www.ncbi.nlm.nih.gov/Taxonomy/Browser/wwwtax.cgi?id=1239) | 52.0 | 1 |
| .......[Staphylococcus aureus subsp. aureus ST398](https://www.ncbi.nlm.nih.gov/Taxonomy/Browser/wwwtax.cgi?id=523796) | [firmicutes](https://www.ncbi.nlm.nih.gov/Taxonomy/Browser/wwwtax.cgi?id=1239) | 52.0 | 6 |
| .......[Staphylococcus hyicus](https://www.ncbi.nlm.nih.gov/Taxonomy/Browser/wwwtax.cgi?id=1284) | [firmicutes](https://www.ncbi.nlm.nih.gov/Taxonomy/Browser/wwwtax.cgi?id=1239) | 52.0 | 2 |
| .......[Staphylococcus aureus subsp. aureus ECT-R 2](https://www.ncbi.nlm.nih.gov/Taxonomy/Browser/wwwtax.cgi?id=889933) | [firmicutes](https://www.ncbi.nlm.nih.gov/Taxonomy/Browser/wwwtax.cgi?id=1239) | 52.0 | 1 |
| .......[Staphylococcus aureus 04-02981](https://www.ncbi.nlm.nih.gov/Taxonomy/Browser/wwwtax.cgi?id=703339) | [firmicutes](https://www.ncbi.nlm.nih.gov/Taxonomy/Browser/wwwtax.cgi?id=1239) | 52.0 | 1 |
| .......[Staphylococcus aureus subsp. aureus str. JKD6008](https://www.ncbi.nlm.nih.gov/Taxonomy/Browser/wwwtax.cgi?id=546342) | [firmicutes](https://www.ncbi.nlm.nih.gov/Taxonomy/Browser/wwwtax.cgi?id=1239) | 52.0 | 1 |
| .......[Staphylococcus aureus subsp. aureus Mu3](https://www.ncbi.nlm.nih.gov/Taxonomy/Browser/wwwtax.cgi?id=418127) | [firmicutes](https://www.ncbi.nlm.nih.gov/Taxonomy/Browser/wwwtax.cgi?id=1239) | 52.0 | 1 |
| .......[Staphylococcus aureus subsp. aureus Mu50](https://www.ncbi.nlm.nih.gov/Taxonomy/Browser/wwwtax.cgi?id=158878) | [firmicutes](https://www.ncbi.nlm.nih.gov/Taxonomy/Browser/wwwtax.cgi?id=1239) | 52.0 | 1 |
| .......[Staphylococcus aureus subsp. aureus N315](https://www.ncbi.nlm.nih.gov/Taxonomy/Browser/wwwtax.cgi?id=158879) | [firmicutes](https://www.ncbi.nlm.nih.gov/Taxonomy/Browser/wwwtax.cgi?id=1239) | 52.0 | 1 |
| .......[Staphylococcus cohnii](https://www.ncbi.nlm.nih.gov/Taxonomy/Browser/wwwtax.cgi?id=29382) | [firmicutes](https://www.ncbi.nlm.nih.gov/Taxonomy/Browser/wwwtax.cgi?id=1239) | 52.0 | 2 |
| .......[Staphylococcus schleiferi](https://www.ncbi.nlm.nih.gov/Taxonomy/Browser/wwwtax.cgi?id=1295) | [firmicutes](https://www.ncbi.nlm.nih.gov/Taxonomy/Browser/wwwtax.cgi?id=1239) | 46.1 | 1 |
| .......[Staphylococcus aureus CA-347](https://www.ncbi.nlm.nih.gov/Taxonomy/Browser/wwwtax.cgi?id=1323661) | [firmicutes](https://www.ncbi.nlm.nih.gov/Taxonomy/Browser/wwwtax.cgi?id=1239) | 44.1 | 1 |
| .......[Staphylococcus saprophyticus](https://www.ncbi.nlm.nih.gov/Taxonomy/Browser/wwwtax.cgi?id=29385) | [firmicutes](https://www.ncbi.nlm.nih.gov/Taxonomy/Browser/wwwtax.cgi?id=1239) | 44.1 | 1 |
| .......[Staphylococcus aureus subsp. aureus MRSA252](https://www.ncbi.nlm.nih.gov/Taxonomy/Browser/wwwtax.cgi?id=282458) | [firmicutes](https://www.ncbi.nlm.nih.gov/Taxonomy/Browser/wwwtax.cgi?id=1239) | 44.1 | 1 |
| ......[Ureibacillus thermosphaericus](https://www.ncbi.nlm.nih.gov/Taxonomy/Browser/wwwtax.cgi?id=51173) | [firmicutes](https://www.ncbi.nlm.nih.gov/Taxonomy/Browser/wwwtax.cgi?id=1239) | 52.0 | 1 |
| ......[Geobacillus stearothermophilus](https://www.ncbi.nlm.nih.gov/Taxonomy/Browser/wwwtax.cgi?id=1422) | [firmicutes](https://www.ncbi.nlm.nih.gov/Taxonomy/Browser/wwwtax.cgi?id=1239) | 52.0 | 1 |
| ......[Bacillus sp.](https://www.ncbi.nlm.nih.gov/Taxonomy/Browser/wwwtax.cgi?id=1409) | [firmicutes](https://www.ncbi.nlm.nih.gov/Taxonomy/Browser/wwwtax.cgi?id=1239) | 52.0 | 2 |
| ......[Bacillus subtilis](https://www.ncbi.nlm.nih.gov/Taxonomy/Browser/wwwtax.cgi?id=1423) | [firmicutes](https://www.ncbi.nlm.nih.gov/Taxonomy/Browser/wwwtax.cgi?id=1239) | 52.0 | 7 |
| ......[Listeria monocytogenes](https://www.ncbi.nlm.nih.gov/Taxonomy/Browser/wwwtax.cgi?id=1639) | [firmicutes](https://www.ncbi.nlm.nih.gov/Taxonomy/Browser/wwwtax.cgi?id=1239) | 52.0 | 1 |
| ......[Bacillus cereus](https://www.ncbi.nlm.nih.gov/Taxonomy/Browser/wwwtax.cgi?id=1396) | [firmicutes](https://www.ncbi.nlm.nih.gov/Taxonomy/Browser/wwwtax.cgi?id=1239) | 52.0 | 1 |
| ......[Bacillus subtilis subsp. subtilis](https://www.ncbi.nlm.nih.gov/Taxonomy/Browser/wwwtax.cgi?id=135461) | [firmicutes](https://www.ncbi.nlm.nih.gov/Taxonomy/Browser/wwwtax.cgi?id=1239) | 38.2 | 1 |
| ......[Bacillus subtilis subsp. inaquosorum](https://www.ncbi.nlm.nih.gov/Taxonomy/Browser/wwwtax.cgi?id=483913) | [firmicutes](https://www.ncbi.nlm.nih.gov/Taxonomy/Browser/wwwtax.cgi?id=1239) | 38.2 | 1 |
| ......[Bacillus sp. YP1](https://www.ncbi.nlm.nih.gov/Taxonomy/Browser/wwwtax.cgi?id=1574141) | [firmicutes](https://www.ncbi.nlm.nih.gov/Taxonomy/Browser/wwwtax.cgi?id=1239) | 38.2 | 1 |
| ......[Bacillus subtilis TO-A](https://www.ncbi.nlm.nih.gov/Taxonomy/Browser/wwwtax.cgi?id=1340494) | [firmicutes](https://www.ncbi.nlm.nih.gov/Taxonomy/Browser/wwwtax.cgi?id=1239) | 38.2 | 1 |
| ......[Bacillus subtilis subsp. subtilis str. RO-NN-1](https://www.ncbi.nlm.nih.gov/Taxonomy/Browser/wwwtax.cgi?id=1052588) | [firmicutes](https://www.ncbi.nlm.nih.gov/Taxonomy/Browser/wwwtax.cgi?id=1239) | 38.2 | 1 |
| ......[Paenibacillus sp. LPB0068](https://www.ncbi.nlm.nih.gov/Taxonomy/Browser/wwwtax.cgi?id=1763538) | [firmicutes](https://www.ncbi.nlm.nih.gov/Taxonomy/Browser/wwwtax.cgi?id=1239) | 36.2 | 1 |
| .....[Lactobacillus salivarius](https://www.ncbi.nlm.nih.gov/Taxonomy/Browser/wwwtax.cgi?id=1624) | [firmicutes](https://www.ncbi.nlm.nih.gov/Taxonomy/Browser/wwwtax.cgi?id=1239) | 52.0 | 2 |
| .....[Enterococcus faecalis](https://www.ncbi.nlm.nih.gov/Taxonomy/Browser/wwwtax.cgi?id=1351) | [firmicutes](https://www.ncbi.nlm.nih.gov/Taxonomy/Browser/wwwtax.cgi?id=1239) | 52.0 | 2 |
| .....[Streptococcus pasteurianus](https://www.ncbi.nlm.nih.gov/Taxonomy/Browser/wwwtax.cgi?id=197614) | [firmicutes](https://www.ncbi.nlm.nih.gov/Taxonomy/Browser/wwwtax.cgi?id=1239) | 52.0 | 1 |
| .....[Enterococcus faecium](https://www.ncbi.nlm.nih.gov/Taxonomy/Browser/wwwtax.cgi?id=1352) | [firmicutes](https://www.ncbi.nlm.nih.gov/Taxonomy/Browser/wwwtax.cgi?id=1239) | 52.0 | 13 |
| .....[Lactobacillus johnsonii](https://www.ncbi.nlm.nih.gov/Taxonomy/Browser/wwwtax.cgi?id=33959) | [firmicutes](https://www.ncbi.nlm.nih.gov/Taxonomy/Browser/wwwtax.cgi?id=1239) | 52.0 | 3 |
| .....[Enterococcus hirae](https://www.ncbi.nlm.nih.gov/Taxonomy/Browser/wwwtax.cgi?id=1354) | [firmicutes](https://www.ncbi.nlm.nih.gov/Taxonomy/Browser/wwwtax.cgi?id=1239) | 52.0 | 1 |
| .....[Lactobacillus reuteri](https://www.ncbi.nlm.nih.gov/Taxonomy/Browser/wwwtax.cgi?id=1598) | [firmicutes](https://www.ncbi.nlm.nih.gov/Taxonomy/Browser/wwwtax.cgi?id=1239) | 52.0 | 2 |
| .....[Enterococcus cecorum](https://www.ncbi.nlm.nih.gov/Taxonomy/Browser/wwwtax.cgi?id=44008) | [firmicutes](https://www.ncbi.nlm.nih.gov/Taxonomy/Browser/wwwtax.cgi?id=1239) | 52.0 | 5 |
| .....[Enterococcus faecium DO](https://www.ncbi.nlm.nih.gov/Taxonomy/Browser/wwwtax.cgi?id=333849) | [firmicutes](https://www.ncbi.nlm.nih.gov/Taxonomy/Browser/wwwtax.cgi?id=1239) | 52.0 | 1 |
| .....[Streptococcus pasteurianus ATCC 43144](https://www.ncbi.nlm.nih.gov/Taxonomy/Browser/wwwtax.cgi?id=981540) | [firmicutes](https://www.ncbi.nlm.nih.gov/Taxonomy/Browser/wwwtax.cgi?id=1239) | 52.0 | 1 |
| .....[Streptococcus gallolyticus UCN34](https://www.ncbi.nlm.nih.gov/Taxonomy/Browser/wwwtax.cgi?id=637909) | [firmicutes](https://www.ncbi.nlm.nih.gov/Taxonomy/Browser/wwwtax.cgi?id=1239) | 52.0 | 1 |
| .....[Streptococcus suis BM407](https://www.ncbi.nlm.nih.gov/Taxonomy/Browser/wwwtax.cgi?id=568814) | [firmicutes](https://www.ncbi.nlm.nih.gov/Taxonomy/Browser/wwwtax.cgi?id=1239) | 52.0 | 1 |
| .....[Streptococcus agalactiae](https://www.ncbi.nlm.nih.gov/Taxonomy/Browser/wwwtax.cgi?id=1311) | [firmicutes](https://www.ncbi.nlm.nih.gov/Taxonomy/Browser/wwwtax.cgi?id=1239) | 52.0 | 5 |
| .....[Streptococcus pyogenes](https://www.ncbi.nlm.nih.gov/Taxonomy/Browser/wwwtax.cgi?id=1314) | [firmicutes](https://www.ncbi.nlm.nih.gov/Taxonomy/Browser/wwwtax.cgi?id=1239) | 44.1 | 2 |
| .....[Streptococcus suis D12](https://www.ncbi.nlm.nih.gov/Taxonomy/Browser/wwwtax.cgi?id=1004952) | [firmicutes](https://www.ncbi.nlm.nih.gov/Taxonomy/Browser/wwwtax.cgi?id=1239) | 44.1 | 1 |
| .....[Streptococcus gallolyticus subsp. gallolyticus ATCC BAA-2069](https://www.ncbi.nlm.nih.gov/Taxonomy/Browser/wwwtax.cgi?id=990317) | [firmicutes](https://www.ncbi.nlm.nih.gov/Taxonomy/Browser/wwwtax.cgi?id=1239) | 42.1 | 1 |
| .....[Streptococcus dysgalactiae subsp. equisimilis](https://www.ncbi.nlm.nih.gov/Taxonomy/Browser/wwwtax.cgi?id=119602) | [firmicutes](https://www.ncbi.nlm.nih.gov/Taxonomy/Browser/wwwtax.cgi?id=1239) | 38.2 | 1 |
| ....[Clostridium beijerinckii](https://www.ncbi.nlm.nih.gov/Taxonomy/Browser/wwwtax.cgi?id=1520) | [firmicutes](https://www.ncbi.nlm.nih.gov/Taxonomy/Browser/wwwtax.cgi?id=1239) | 36.2 | 1 |
| ...[uncultured bacterium](https://www.ncbi.nlm.nih.gov/Taxonomy/Browser/wwwtax.cgi?id=77133) | [bacteria](https://www.ncbi.nlm.nih.gov/Taxonomy/Browser/wwwtax.cgi?id=-1) | 52.0 | 15 |
| ...[Escherichia coli](https://www.ncbi.nlm.nih.gov/Taxonomy/Browser/wwwtax.cgi?id=562) | [enterobacteria](https://www.ncbi.nlm.nih.gov/Taxonomy/Browser/wwwtax.cgi?id=91347) | 52.0 | 3 |
| ...[Proteus vulgaris](https://www.ncbi.nlm.nih.gov/Taxonomy/Browser/wwwtax.cgi?id=585) | [enterobacteria](https://www.ncbi.nlm.nih.gov/Taxonomy/Browser/wwwtax.cgi?id=91347) | 52.0 | 1 |
| ...[uncultured bacterium MID12](https://www.ncbi.nlm.nih.gov/Taxonomy/Browser/wwwtax.cgi?id=764346) | [bacteria](https://www.ncbi.nlm.nih.gov/Taxonomy/Browser/wwwtax.cgi?id=-1) | 52.0 | 1 |
| ...[uncultured bacterium IN-07](https://www.ncbi.nlm.nih.gov/Taxonomy/Browser/wwwtax.cgi?id=1805585) | [bacteria](https://www.ncbi.nlm.nih.gov/Taxonomy/Browser/wwwtax.cgi?id=-1) | 42.1 | 1 |
| ...[Bernardetia litoralis DSM 6794](https://www.ncbi.nlm.nih.gov/Taxonomy/Browser/wwwtax.cgi?id=880071) | [CFB group bacteria](https://www.ncbi.nlm.nih.gov/Taxonomy/Browser/wwwtax.cgi?id=976) | 38.2 | 1 |
| ...[uncultured Epsilonproteobacteria bacterium](https://www.ncbi.nlm.nih.gov/Taxonomy/Browser/wwwtax.cgi?id=120858) | [e-proteobacteria](https://www.ncbi.nlm.nih.gov/Taxonomy/Browser/wwwtax.cgi?id=29547) | 38.2 | 1 |
| ...[Cyclobacterium marinum DSM 745](https://www.ncbi.nlm.nih.gov/Taxonomy/Browser/wwwtax.cgi?id=880070) | [CFB group bacteria](https://www.ncbi.nlm.nih.gov/Taxonomy/Browser/wwwtax.cgi?id=976) | 36.2 | 1 |
| ..[Homo sapiens](https://www.ncbi.nlm.nih.gov/Taxonomy/Browser/wwwtax.cgi?id=9606) | [primates](https://www.ncbi.nlm.nih.gov/Taxonomy/Browser/wwwtax.cgi?id=9443) | 42.1 | 9 |
| ..[Apteryx australis mantelli](https://www.ncbi.nlm.nih.gov/Taxonomy/Browser/wwwtax.cgi?id=202946) | [birds](https://www.ncbi.nlm.nih.gov/Taxonomy/Browser/wwwtax.cgi?id=8782) | 42.1 | 1 |
| ..[Schistosoma margrebowiei](https://www.ncbi.nlm.nih.gov/Taxonomy/Browser/wwwtax.cgi?id=48269) | [flatworms](https://www.ncbi.nlm.nih.gov/Taxonomy/Browser/wwwtax.cgi?id=6157) | 42.1 | 3 |
| ..[Lupinus angustiflorus](https://www.ncbi.nlm.nih.gov/Taxonomy/Browser/wwwtax.cgi?id=749240) | [eudicots](https://www.ncbi.nlm.nih.gov/Taxonomy/Browser/wwwtax.cgi?id=71240) | 40.1 | 4 |
| ..[Oryzias latipes](https://www.ncbi.nlm.nih.gov/Taxonomy/Browser/wwwtax.cgi?id=8090) | [bony fishes](https://www.ncbi.nlm.nih.gov/Taxonomy/Browser/wwwtax.cgi?id=7898) | 40.1 | 6 |
| ..[Helianthus annuus](https://www.ncbi.nlm.nih.gov/Taxonomy/Browser/wwwtax.cgi?id=4232) | [eudicots](https://www.ncbi.nlm.nih.gov/Taxonomy/Browser/wwwtax.cgi?id=71240) | 38.2 | 1 |
| ..[Sesamum indicum](https://www.ncbi.nlm.nih.gov/Taxonomy/Browser/wwwtax.cgi?id=4182) | [eudicots](https://www.ncbi.nlm.nih.gov/Taxonomy/Browser/wwwtax.cgi?id=71240) | 38.2 | 1 |
| ..[Pleurospermum camtschaticum](https://www.ncbi.nlm.nih.gov/Taxonomy/Browser/wwwtax.cgi?id=49565) | [eudicots](https://www.ncbi.nlm.nih.gov/Taxonomy/Browser/wwwtax.cgi?id=71240) | 38.2 | 1 |
| ..[Leishmania tarentolae](https://www.ncbi.nlm.nih.gov/Taxonomy/Browser/wwwtax.cgi?id=5689) | [kinetoplastids](https://www.ncbi.nlm.nih.gov/Taxonomy/Browser/wwwtax.cgi?id=5653) | 38.2 | 5 |
| ..[Sus scrofa](https://www.ncbi.nlm.nih.gov/Taxonomy/Browser/wwwtax.cgi?id=9823) | [even-toed ungulates](https://www.ncbi.nlm.nih.gov/Taxonomy/Browser/wwwtax.cgi?id=91561) | 38.2 | 4 |
| ..[Erinaceus europaeus](https://www.ncbi.nlm.nih.gov/Taxonomy/Browser/wwwtax.cgi?id=9365) | [insectivores](https://www.ncbi.nlm.nih.gov/Taxonomy/Browser/wwwtax.cgi?id=9362) | 38.2 | 1 |
| ..[Vigna angularis var. angularis](https://www.ncbi.nlm.nih.gov/Taxonomy/Browser/wwwtax.cgi?id=157739) | [eudicots](https://www.ncbi.nlm.nih.gov/Taxonomy/Browser/wwwtax.cgi?id=71240) | 38.2 | 2 |
| ..[Enterobius vermicularis](https://www.ncbi.nlm.nih.gov/Taxonomy/Browser/wwwtax.cgi?id=51028) | [nematodes](https://www.ncbi.nlm.nih.gov/Taxonomy/Browser/wwwtax.cgi?id=-1) | 38.2 | 1 |
| ..[Cyprinus carpio](https://www.ncbi.nlm.nih.gov/Taxonomy/Browser/wwwtax.cgi?id=7962) | [bony fishes](https://www.ncbi.nlm.nih.gov/Taxonomy/Browser/wwwtax.cgi?id=7898) | 38.2 | 6 |
| ..[Schistosoma mansoni](https://www.ncbi.nlm.nih.gov/Taxonomy/Browser/wwwtax.cgi?id=6183) | [flatworms](https://www.ncbi.nlm.nih.gov/Taxonomy/Browser/wwwtax.cgi?id=6157) | 38.2 | 2 |
| ..[Anthriscus cerefolium](https://www.ncbi.nlm.nih.gov/Taxonomy/Browser/wwwtax.cgi?id=40888) | [eudicots](https://www.ncbi.nlm.nih.gov/Taxonomy/Browser/wwwtax.cgi?id=71240) | 38.2 | 1 |
| ..[Populus trichocarpa](https://www.ncbi.nlm.nih.gov/Taxonomy/Browser/wwwtax.cgi?id=3694) | [eudicots](https://www.ncbi.nlm.nih.gov/Taxonomy/Browser/wwwtax.cgi?id=71240) | 38.2 | 1 |
| ..[Vitis vinifera](https://www.ncbi.nlm.nih.gov/Taxonomy/Browser/wwwtax.cgi?id=29760) | [eudicots](https://www.ncbi.nlm.nih.gov/Taxonomy/Browser/wwwtax.cgi?id=71240) | 38.2 | 2 |
| ..[Medicago truncatula](https://www.ncbi.nlm.nih.gov/Taxonomy/Browser/wwwtax.cgi?id=3880) | [eudicots](https://www.ncbi.nlm.nih.gov/Taxonomy/Browser/wwwtax.cgi?id=71240) | 38.2 | 2 |
| ..[Danio rerio](https://www.ncbi.nlm.nih.gov/Taxonomy/Browser/wwwtax.cgi?id=7955) | [bony fishes](https://www.ncbi.nlm.nih.gov/Taxonomy/Browser/wwwtax.cgi?id=7898) | 38.2 | 9 |
| ..[Laccotrephes japonensis](https://www.ncbi.nlm.nih.gov/Taxonomy/Browser/wwwtax.cgi?id=500712) | [bugs](https://www.ncbi.nlm.nih.gov/Taxonomy/Browser/wwwtax.cgi?id=7524) | 36.2 | 2 |
| ..[Laccotrephes sp. JRR-2017](https://www.ncbi.nlm.nih.gov/Taxonomy/Browser/wwwtax.cgi?id=2034366) | [bugs](https://www.ncbi.nlm.nih.gov/Taxonomy/Browser/wwwtax.cgi?id=7524) | 36.2 | 1 |
| ..[Papio anubis](https://www.ncbi.nlm.nih.gov/Taxonomy/Browser/wwwtax.cgi?id=9555) | [primates](https://www.ncbi.nlm.nih.gov/Taxonomy/Browser/wwwtax.cgi?id=9443) | 36.2 | 10 |
| ..[Placobdella phalera](https://www.ncbi.nlm.nih.gov/Taxonomy/Browser/wwwtax.cgi?id=60955) | [segmented worms](https://www.ncbi.nlm.nih.gov/Taxonomy/Browser/wwwtax.cgi?id=6340) | 36.2 | 4 |
| ..[Manihot esculenta](https://www.ncbi.nlm.nih.gov/Taxonomy/Browser/wwwtax.cgi?id=3983) | [eudicots](https://www.ncbi.nlm.nih.gov/Taxonomy/Browser/wwwtax.cgi?id=71240) | 36.2 | 3 |
| ..[Tingidae sp. BIOUG04904-E06](https://www.ncbi.nlm.nih.gov/Taxonomy/Browser/wwwtax.cgi?id=2002681) | [bugs](https://www.ncbi.nlm.nih.gov/Taxonomy/Browser/wwwtax.cgi?id=7524) | 36.2 | 1 |
| ..[Tingidae sp. BIOUG04904-D11](https://www.ncbi.nlm.nih.gov/Taxonomy/Browser/wwwtax.cgi?id=2002680) | [bugs](https://www.ncbi.nlm.nih.gov/Taxonomy/Browser/wwwtax.cgi?id=7524) | 36.2 | 1 |
| ..[Tingidae sp. BIOUG02579-C06](https://www.ncbi.nlm.nih.gov/Taxonomy/Browser/wwwtax.cgi?id=2002679) | [bugs](https://www.ncbi.nlm.nih.gov/Taxonomy/Browser/wwwtax.cgi?id=7524) | 36.2 | 1 |
| ..[Tingidae sp. BIOUG02579-C08](https://www.ncbi.nlm.nih.gov/Taxonomy/Browser/wwwtax.cgi?id=2002678) | [bugs](https://www.ncbi.nlm.nih.gov/Taxonomy/Browser/wwwtax.cgi?id=7524) | 36.2 | 1 |
| ..[Tingidae sp. BIOUG02579-C07](https://www.ncbi.nlm.nih.gov/Taxonomy/Browser/wwwtax.cgi?id=2002677) | [bugs](https://www.ncbi.nlm.nih.gov/Taxonomy/Browser/wwwtax.cgi?id=7524) | 36.2 | 1 |
| ..[Hansenia forrestii](https://www.ncbi.nlm.nih.gov/Taxonomy/Browser/wwwtax.cgi?id=1050841) | [eudicots](https://www.ncbi.nlm.nih.gov/Taxonomy/Browser/wwwtax.cgi?id=71240) | 36.2 | 1 |
| ..[Hansenia oviformis](https://www.ncbi.nlm.nih.gov/Taxonomy/Browser/wwwtax.cgi?id=1978917) | [eudicots](https://www.ncbi.nlm.nih.gov/Taxonomy/Browser/wwwtax.cgi?id=71240) | 36.2 | 1 |
| ..[Hansenia forbesii](https://www.ncbi.nlm.nih.gov/Taxonomy/Browser/wwwtax.cgi?id=165499) | [eudicots](https://www.ncbi.nlm.nih.gov/Taxonomy/Browser/wwwtax.cgi?id=71240) | 36.2 | 1 |
| ..[Hansenia weberbaueriana](https://www.ncbi.nlm.nih.gov/Taxonomy/Browser/wwwtax.cgi?id=54724) | [eudicots](https://www.ncbi.nlm.nih.gov/Taxonomy/Browser/wwwtax.cgi?id=71240) | 36.2 | 1 |
| ..[Tingidae sp. BIOUG04904-F05](https://www.ncbi.nlm.nih.gov/Taxonomy/Browser/wwwtax.cgi?id=1996501) | [bugs](https://www.ncbi.nlm.nih.gov/Taxonomy/Browser/wwwtax.cgi?id=7524) | 36.2 | 1 |
| ..[Heterocephalus glaber](https://www.ncbi.nlm.nih.gov/Taxonomy/Browser/wwwtax.cgi?id=10181) | [rodents](https://www.ncbi.nlm.nih.gov/Taxonomy/Browser/wwwtax.cgi?id=9989) | 36.2 | 6 |
| ..[Mesocricetus auratus](https://www.ncbi.nlm.nih.gov/Taxonomy/Browser/wwwtax.cgi?id=10036) | [rodents](https://www.ncbi.nlm.nih.gov/Taxonomy/Browser/wwwtax.cgi?id=9989) | 36.2 | 4 |
| ..[Mus pahari](https://www.ncbi.nlm.nih.gov/Taxonomy/Browser/wwwtax.cgi?id=10093) | [rodents](https://www.ncbi.nlm.nih.gov/Taxonomy/Browser/wwwtax.cgi?id=9989) | 36.2 | 3 |
| ..[Peucedanum japonicum](https://www.ncbi.nlm.nih.gov/Taxonomy/Browser/wwwtax.cgi?id=49563) | [eudicots](https://www.ncbi.nlm.nih.gov/Taxonomy/Browser/wwwtax.cgi?id=71240) | 36.2 | 2 |
| ..[Ledebouriella seseloides](https://www.ncbi.nlm.nih.gov/Taxonomy/Browser/wwwtax.cgi?id=656325) | [eudicots](https://www.ncbi.nlm.nih.gov/Taxonomy/Browser/wwwtax.cgi?id=71240) | 36.2 | 2 |
| ..[Oncorhynchus kisutch](https://www.ncbi.nlm.nih.gov/Taxonomy/Browser/wwwtax.cgi?id=8019) | [bony fishes](https://www.ncbi.nlm.nih.gov/Taxonomy/Browser/wwwtax.cgi?id=7898) | 36.2 | 3 |
| ..[Loa loa](https://www.ncbi.nlm.nih.gov/Taxonomy/Browser/wwwtax.cgi?id=7209) | [nematodes](https://www.ncbi.nlm.nih.gov/Taxonomy/Browser/wwwtax.cgi?id=-1) | 36.2 | 1 |
| ..[Caucalis platycarpos](https://www.ncbi.nlm.nih.gov/Taxonomy/Browser/wwwtax.cgi?id=52456) | [eudicots](https://www.ncbi.nlm.nih.gov/Taxonomy/Browser/wwwtax.cgi?id=71240) | 36.2 | 1 |
| ..[Daucus conchitae](https://www.ncbi.nlm.nih.gov/Taxonomy/Browser/wwwtax.cgi?id=180803) | [eudicots](https://www.ncbi.nlm.nih.gov/Taxonomy/Browser/wwwtax.cgi?id=71240) | 36.2 | 1 |
| ..[Daucus pusillus](https://www.ncbi.nlm.nih.gov/Taxonomy/Browser/wwwtax.cgi?id=79171) | [eudicots](https://www.ncbi.nlm.nih.gov/Taxonomy/Browser/wwwtax.cgi?id=71240) | 36.2 | 2 |
| ..[Daucus setulosus](https://www.ncbi.nlm.nih.gov/Taxonomy/Browser/wwwtax.cgi?id=1792663) | [eudicots](https://www.ncbi.nlm.nih.gov/Taxonomy/Browser/wwwtax.cgi?id=71240) | 36.2 | 2 |
| ..[Daucus guttatus](https://www.ncbi.nlm.nih.gov/Taxonomy/Browser/wwwtax.cgi?id=79165) | [eudicots](https://www.ncbi.nlm.nih.gov/Taxonomy/Browser/wwwtax.cgi?id=71240) | 36.2 | 4 |
| ..[Daucus glochidiatus](https://www.ncbi.nlm.nih.gov/Taxonomy/Browser/wwwtax.cgi?id=180807) | [eudicots](https://www.ncbi.nlm.nih.gov/Taxonomy/Browser/wwwtax.cgi?id=71240) | 36.2 | 1 |
| ..[Daucus littoralis](https://www.ncbi.nlm.nih.gov/Taxonomy/Browser/wwwtax.cgi?id=571607) | [eudicots](https://www.ncbi.nlm.nih.gov/Taxonomy/Browser/wwwtax.cgi?id=71240) | 36.2 | 1 |
| ..[Daucus tenuisectus](https://www.ncbi.nlm.nih.gov/Taxonomy/Browser/wwwtax.cgi?id=180800) | [eudicots](https://www.ncbi.nlm.nih.gov/Taxonomy/Browser/wwwtax.cgi?id=71240) | 36.2 | 1 |
| ..[Daucus crinitus](https://www.ncbi.nlm.nih.gov/Taxonomy/Browser/wwwtax.cgi?id=79167) | [eudicots](https://www.ncbi.nlm.nih.gov/Taxonomy/Browser/wwwtax.cgi?id=71240) | 36.2 | 2 |
| ..[Daucus muricatus](https://www.ncbi.nlm.nih.gov/Taxonomy/Browser/wwwtax.cgi?id=79170) | [eudicots](https://www.ncbi.nlm.nih.gov/Taxonomy/Browser/wwwtax.cgi?id=71240) | 36.2 | 2 |
| ..[Daucus aureus](https://www.ncbi.nlm.nih.gov/Taxonomy/Browser/wwwtax.cgi?id=79164) | [eudicots](https://www.ncbi.nlm.nih.gov/Taxonomy/Browser/wwwtax.cgi?id=71240) | 36.2 | 1 |
| ..[Daucus pumilus](https://www.ncbi.nlm.nih.gov/Taxonomy/Browser/wwwtax.cgi?id=40858) | [eudicots](https://www.ncbi.nlm.nih.gov/Taxonomy/Browser/wwwtax.cgi?id=71240) | 36.2 | 1 |
| ..[Daucus rouyi](https://www.ncbi.nlm.nih.gov/Taxonomy/Browser/wwwtax.cgi?id=571641) | [eudicots](https://www.ncbi.nlm.nih.gov/Taxonomy/Browser/wwwtax.cgi?id=71240) | 36.2 | 1 |
| ..[Cuniculiplasma divulgatum](https://www.ncbi.nlm.nih.gov/Taxonomy/Browser/wwwtax.cgi?id=1673428) | [euryarchaeotes](https://www.ncbi.nlm.nih.gov/Taxonomy/Browser/wwwtax.cgi?id=28890) | 36.2 | 2 |
| ..[Spirodela polyrhiza](https://www.ncbi.nlm.nih.gov/Taxonomy/Browser/wwwtax.cgi?id=29656) | [monocots](https://www.ncbi.nlm.nih.gov/Taxonomy/Browser/wwwtax.cgi?id=4447) | 36.2 | 1 |
| ..[Peucedanum insolens](https://www.ncbi.nlm.nih.gov/Taxonomy/Browser/wwwtax.cgi?id=1680231) | [eudicots](https://www.ncbi.nlm.nih.gov/Taxonomy/Browser/wwwtax.cgi?id=71240) | 36.2 | 1 |
| ..[Lytorhynchus diadema](https://www.ncbi.nlm.nih.gov/Taxonomy/Browser/wwwtax.cgi?id=224560) | [snakes](https://www.ncbi.nlm.nih.gov/Taxonomy/Browser/wwwtax.cgi?id=8570) | 36.2 | 1 |
| ..[Arracacia xanthorrhiza](https://www.ncbi.nlm.nih.gov/Taxonomy/Browser/wwwtax.cgi?id=264540) | [eudicots](https://www.ncbi.nlm.nih.gov/Taxonomy/Browser/wwwtax.cgi?id=71240) | 36.2 | 2 |
| ..[Oreochromis niloticus](https://www.ncbi.nlm.nih.gov/Taxonomy/Browser/wwwtax.cgi?id=8128) | [bony fishes](https://www.ncbi.nlm.nih.gov/Taxonomy/Browser/wwwtax.cgi?id=7898) | 36.2 | 3 |
| ..[Kwoniella pini CBS 10737](https://www.ncbi.nlm.nih.gov/Taxonomy/Browser/wwwtax.cgi?id=1296096) | [basidiomycetes](https://www.ncbi.nlm.nih.gov/Taxonomy/Browser/wwwtax.cgi?id=5204) | 36.2 | 1 |
| ..[Ciona intestinalis](https://www.ncbi.nlm.nih.gov/Taxonomy/Browser/wwwtax.cgi?id=7719) | [tunicates](https://www.ncbi.nlm.nih.gov/Taxonomy/Browser/wwwtax.cgi?id=7712) | 36.2 | 2 |
| ..[Plasmodium gaboni](https://www.ncbi.nlm.nih.gov/Taxonomy/Browser/wwwtax.cgi?id=647221) | [apicomplexans](https://www.ncbi.nlm.nih.gov/Taxonomy/Browser/wwwtax.cgi?id=5794) | 36.2 | 1 |
| ..[Plasmodium falciparum 3D7](https://www.ncbi.nlm.nih.gov/Taxonomy/Browser/wwwtax.cgi?id=36329) | [apicomplexans](https://www.ncbi.nlm.nih.gov/Taxonomy/Browser/wwwtax.cgi?id=5794) | 36.2 | 3 |
| ..[Rhinopithecus bieti](https://www.ncbi.nlm.nih.gov/Taxonomy/Browser/wwwtax.cgi?id=61621) | [primates](https://www.ncbi.nlm.nih.gov/Taxonomy/Browser/wwwtax.cgi?id=9443) | 36.2 | 3 |
| ..[Manis javanica](https://www.ncbi.nlm.nih.gov/Taxonomy/Browser/wwwtax.cgi?id=9974) | [placentals](https://www.ncbi.nlm.nih.gov/Taxonomy/Browser/wwwtax.cgi?id=9347) | 36.2 | 2 |
| ..[Rattus norvegicus](https://www.ncbi.nlm.nih.gov/Taxonomy/Browser/wwwtax.cgi?id=10116) | [rodents](https://www.ncbi.nlm.nih.gov/Taxonomy/Browser/wwwtax.cgi?id=9989) | 36.2 | 6 |
| ..[Theobroma cacao](https://www.ncbi.nlm.nih.gov/Taxonomy/Browser/wwwtax.cgi?id=3641) | [eudicots](https://www.ncbi.nlm.nih.gov/Taxonomy/Browser/wwwtax.cgi?id=71240) | 36.2 | 1 |
| ..[Heleomyzidae sp. BOLD-2016](https://www.ncbi.nlm.nih.gov/Taxonomy/Browser/wwwtax.cgi?id=1886958) | [flies](https://www.ncbi.nlm.nih.gov/Taxonomy/Browser/wwwtax.cgi?id=7147) | 36.2 | 1 |
| ..[Cantharidae sp. BOLD:ACN5366](https://www.ncbi.nlm.nih.gov/Taxonomy/Browser/wwwtax.cgi?id=1770775) | [beetles](https://www.ncbi.nlm.nih.gov/Taxonomy/Browser/wwwtax.cgi?id=7041) | 36.2 | 1 |
| ..[Cantharidae sp. BOLD:ACL8343](https://www.ncbi.nlm.nih.gov/Taxonomy/Browser/wwwtax.cgi?id=1770773) | [beetles](https://www.ncbi.nlm.nih.gov/Taxonomy/Browser/wwwtax.cgi?id=7041) | 36.2 | 17 |
| ..[Carum carvi](https://www.ncbi.nlm.nih.gov/Taxonomy/Browser/wwwtax.cgi?id=48032) | [eudicots](https://www.ncbi.nlm.nih.gov/Taxonomy/Browser/wwwtax.cgi?id=71240) | 36.2 | 1 |
| ..[Anethum graveolens](https://www.ncbi.nlm.nih.gov/Taxonomy/Browser/wwwtax.cgi?id=40922) | [eudicots](https://www.ncbi.nlm.nih.gov/Taxonomy/Browser/wwwtax.cgi?id=71240) | 36.2 | 1 |
| ..[Foeniculum vulgare](https://www.ncbi.nlm.nih.gov/Taxonomy/Browser/wwwtax.cgi?id=48038) | [eudicots](https://www.ncbi.nlm.nih.gov/Taxonomy/Browser/wwwtax.cgi?id=71240) | 36.2 | 1 |
| ..[Macaca fascicularis](https://www.ncbi.nlm.nih.gov/Taxonomy/Browser/wwwtax.cgi?id=9541) | [primates](https://www.ncbi.nlm.nih.gov/Taxonomy/Browser/wwwtax.cgi?id=9443) | 36.2 | 17 |
| ..[Angelica acutiloba](https://www.ncbi.nlm.nih.gov/Taxonomy/Browser/wwwtax.cgi?id=55605) | [eudicots](https://www.ncbi.nlm.nih.gov/Taxonomy/Browser/wwwtax.cgi?id=71240) | 36.2 | 1 |
| ..[Macaca mulatta](https://www.ncbi.nlm.nih.gov/Taxonomy/Browser/wwwtax.cgi?id=9544) | [primates](https://www.ncbi.nlm.nih.gov/Taxonomy/Browser/wwwtax.cgi?id=9443) | 36.2 | 6 |
| ..[Equus asinus](https://www.ncbi.nlm.nih.gov/Taxonomy/Browser/wwwtax.cgi?id=9793) | [odd-toed ungulates](https://www.ncbi.nlm.nih.gov/Taxonomy/Browser/wwwtax.cgi?id=9787) | 36.2 | 1 |
| ..[Ceratotherium simum simum](https://www.ncbi.nlm.nih.gov/Taxonomy/Browser/wwwtax.cgi?id=73337) | [odd-toed ungulates](https://www.ncbi.nlm.nih.gov/Taxonomy/Browser/wwwtax.cgi?id=9787) | 36.2 | 1 |
| ..[Equus caballus](https://www.ncbi.nlm.nih.gov/Taxonomy/Browser/wwwtax.cgi?id=9796) | [odd-toed ungulates](https://www.ncbi.nlm.nih.gov/Taxonomy/Browser/wwwtax.cgi?id=9787) | 36.2 | 1 |
| ..[Heleomyzidae sp. BOLD:ACD1713](https://www.ncbi.nlm.nih.gov/Taxonomy/Browser/wwwtax.cgi?id=1694008) | [flies](https://www.ncbi.nlm.nih.gov/Taxonomy/Browser/wwwtax.cgi?id=7147) | 36.2 | 6 |
| ..[Tuberculefferia producta](https://www.ncbi.nlm.nih.gov/Taxonomy/Browser/wwwtax.cgi?id=1717641) | [flies](https://www.ncbi.nlm.nih.gov/Taxonomy/Browser/wwwtax.cgi?id=7147) | 36.2 | 1 |
| ..[Glossiphoniidae sp. BOLD:AAF9645](https://www.ncbi.nlm.nih.gov/Taxonomy/Browser/wwwtax.cgi?id=1651980) | [segmented worms](https://www.ncbi.nlm.nih.gov/Taxonomy/Browser/wwwtax.cgi?id=6340) | 36.2 | 1 |
| ..[Platygastridae sp. BOLD:ACJ2438](https://www.ncbi.nlm.nih.gov/Taxonomy/Browser/wwwtax.cgi?id=1669723) | [wasps &c.](https://www.ncbi.nlm.nih.gov/Taxonomy/Browser/wwwtax.cgi?id=7400) | 36.2 | 1 |
| ..[Trichoderma atroviride IMI 206040](https://www.ncbi.nlm.nih.gov/Taxonomy/Browser/wwwtax.cgi?id=452589) | [ascomycetes](https://www.ncbi.nlm.nih.gov/Taxonomy/Browser/wwwtax.cgi?id=4890) | 36.2 | 1 |
| ..[Amyelois transitella](https://www.ncbi.nlm.nih.gov/Taxonomy/Browser/wwwtax.cgi?id=680683) | [moths](https://www.ncbi.nlm.nih.gov/Taxonomy/Browser/wwwtax.cgi?id=7088) | 36.2 | 1 |
| ..[Seseli montanum](https://www.ncbi.nlm.nih.gov/Taxonomy/Browser/wwwtax.cgi?id=40952) | [eudicots](https://www.ncbi.nlm.nih.gov/Taxonomy/Browser/wwwtax.cgi?id=71240) | 36.2 | 1 |
| ..[Pastinaca pimpinellifolia](https://www.ncbi.nlm.nih.gov/Taxonomy/Browser/wwwtax.cgi?id=376870) | [eudicots](https://www.ncbi.nlm.nih.gov/Taxonomy/Browser/wwwtax.cgi?id=71240) | 36.2 | 1 |
| ..[Ovis canadensis canadensis](https://www.ncbi.nlm.nih.gov/Taxonomy/Browser/wwwtax.cgi?id=112262) | [even-toed ungulates](https://www.ncbi.nlm.nih.gov/Taxonomy/Browser/wwwtax.cgi?id=91561) | 36.2 | 7 |
| ..[Plasmodium reichenowi](https://www.ncbi.nlm.nih.gov/Taxonomy/Browser/wwwtax.cgi?id=5854) | [apicomplexans](https://www.ncbi.nlm.nih.gov/Taxonomy/Browser/wwwtax.cgi?id=5794) | 36.2 | 1 |
| ..[Linepithema humile](https://www.ncbi.nlm.nih.gov/Taxonomy/Browser/wwwtax.cgi?id=83485) | [ants](https://www.ncbi.nlm.nih.gov/Taxonomy/Browser/wwwtax.cgi?id=36668) | 36.2 | 1 |
| ..[Nemopoda mamaevi](https://www.ncbi.nlm.nih.gov/Taxonomy/Browser/wwwtax.cgi?id=1577136) | [flies](https://www.ncbi.nlm.nih.gov/Taxonomy/Browser/wwwtax.cgi?id=7147) | 36.2 | 1 |
| ..[Macaca nemestrina](https://www.ncbi.nlm.nih.gov/Taxonomy/Browser/wwwtax.cgi?id=9545) | [primates](https://www.ncbi.nlm.nih.gov/Taxonomy/Browser/wwwtax.cgi?id=9443) | 36.2 | 8 |
| ..[Cercocebus atys](https://www.ncbi.nlm.nih.gov/Taxonomy/Browser/wwwtax.cgi?id=9531) | [primates](https://www.ncbi.nlm.nih.gov/Taxonomy/Browser/wwwtax.cgi?id=9443) | 36.2 | 7 |
| ..[Colobus angolensis palliatus](https://www.ncbi.nlm.nih.gov/Taxonomy/Browser/wwwtax.cgi?id=336983) | [primates](https://www.ncbi.nlm.nih.gov/Taxonomy/Browser/wwwtax.cgi?id=9443) | 36.2 | 4 |
| ..[Mandrillus leucophaeus](https://www.ncbi.nlm.nih.gov/Taxonomy/Browser/wwwtax.cgi?id=9568) | [primates](https://www.ncbi.nlm.nih.gov/Taxonomy/Browser/wwwtax.cgi?id=9443) | 36.2 | 3 |
| ..[Fragaria vesca subsp. vesca](https://www.ncbi.nlm.nih.gov/Taxonomy/Browser/wwwtax.cgi?id=101020) | [eudicots](https://www.ncbi.nlm.nih.gov/Taxonomy/Browser/wwwtax.cgi?id=71240) | 36.2 | 2 |
| ..[Laccotrephes maculatus](https://www.ncbi.nlm.nih.gov/Taxonomy/Browser/wwwtax.cgi?id=1586135) | [bugs](https://www.ncbi.nlm.nih.gov/Taxonomy/Browser/wwwtax.cgi?id=7524) | 36.2 | 1 |
| ..[Hemiclepsis marginata](https://www.ncbi.nlm.nih.gov/Taxonomy/Browser/wwwtax.cgi?id=60929) | [segmented worms](https://www.ncbi.nlm.nih.gov/Taxonomy/Browser/wwwtax.cgi?id=6340) | 36.2 | 2 |
| ..[Malthodes spathifer](https://www.ncbi.nlm.nih.gov/Taxonomy/Browser/wwwtax.cgi?id=1553555) | [beetles](https://www.ncbi.nlm.nih.gov/Taxonomy/Browser/wwwtax.cgi?id=7041) | 36.2 | 6 |
| ..[Rhinopithecus roxellana](https://www.ncbi.nlm.nih.gov/Taxonomy/Browser/wwwtax.cgi?id=61622) | [primates](https://www.ncbi.nlm.nih.gov/Taxonomy/Browser/wwwtax.cgi?id=9443) | 36.2 | 1 |
| ..[Protopolystoma xenopodis](https://www.ncbi.nlm.nih.gov/Taxonomy/Browser/wwwtax.cgi?id=117903) | [flatworms](https://www.ncbi.nlm.nih.gov/Taxonomy/Browser/wwwtax.cgi?id=6157) | 36.2 | 1 |
| ..[Schistosoma mattheei](https://www.ncbi.nlm.nih.gov/Taxonomy/Browser/wwwtax.cgi?id=31246) | [flatworms](https://www.ncbi.nlm.nih.gov/Taxonomy/Browser/wwwtax.cgi?id=6157) | 36.2 | 1 |
| ..[Schistosoma curassoni](https://www.ncbi.nlm.nih.gov/Taxonomy/Browser/wwwtax.cgi?id=6186) | [flatworms](https://www.ncbi.nlm.nih.gov/Taxonomy/Browser/wwwtax.cgi?id=6157) | 36.2 | 1 |
| ..[Strongyloides stercoralis](https://www.ncbi.nlm.nih.gov/Taxonomy/Browser/wwwtax.cgi?id=6248) | [nematodes](https://www.ncbi.nlm.nih.gov/Taxonomy/Browser/wwwtax.cgi?id=-1) | 36.2 | 1 |
| ..[Schistosoma rodhaini](https://www.ncbi.nlm.nih.gov/Taxonomy/Browser/wwwtax.cgi?id=6188) | [flatworms](https://www.ncbi.nlm.nih.gov/Taxonomy/Browser/wwwtax.cgi?id=6157) | 36.2 | 1 |
| ..[Schistocephalus solidus](https://www.ncbi.nlm.nih.gov/Taxonomy/Browser/wwwtax.cgi?id=70667) | [flatworms](https://www.ncbi.nlm.nih.gov/Taxonomy/Browser/wwwtax.cgi?id=6157) | 36.2 | 1 |
| ..[Diphyllobothrium latum](https://www.ncbi.nlm.nih.gov/Taxonomy/Browser/wwwtax.cgi?id=60516) | [flatworms](https://www.ncbi.nlm.nih.gov/Taxonomy/Browser/wwwtax.cgi?id=6157) | 36.2 | 2 |
| ..[Echinostoma caproni](https://www.ncbi.nlm.nih.gov/Taxonomy/Browser/wwwtax.cgi?id=27848) | [flatworms](https://www.ncbi.nlm.nih.gov/Taxonomy/Browser/wwwtax.cgi?id=6157) | 36.2 | 1 |
| ..[Trichobilharzia regenti](https://www.ncbi.nlm.nih.gov/Taxonomy/Browser/wwwtax.cgi?id=157069) | [flatworms](https://www.ncbi.nlm.nih.gov/Taxonomy/Browser/wwwtax.cgi?id=6157) | 36.2 | 1 |
| ..[Caenorhabditis elegans](https://www.ncbi.nlm.nih.gov/Taxonomy/Browser/wwwtax.cgi?id=6239) | [nematodes](https://www.ncbi.nlm.nih.gov/Taxonomy/Browser/wwwtax.cgi?id=-1) | 36.2 | 5 |
| ..[Simalia kinghorni](https://www.ncbi.nlm.nih.gov/Taxonomy/Browser/wwwtax.cgi?id=129330) | [snakes](https://www.ncbi.nlm.nih.gov/Taxonomy/Browser/wwwtax.cgi?id=8570) | 36.2 | 1 |
| ..[Equus przewalskii](https://www.ncbi.nlm.nih.gov/Taxonomy/Browser/wwwtax.cgi?id=9798) | [odd-toed ungulates](https://www.ncbi.nlm.nih.gov/Taxonomy/Browser/wwwtax.cgi?id=9787) | 36.2 | 1 |
| ..[Solanum lycopersicum](https://www.ncbi.nlm.nih.gov/Taxonomy/Browser/wwwtax.cgi?id=4081) | [eudicots](https://www.ncbi.nlm.nih.gov/Taxonomy/Browser/wwwtax.cgi?id=71240) | 36.2 | 3 |
| ..[Solanum pennellii](https://www.ncbi.nlm.nih.gov/Taxonomy/Browser/wwwtax.cgi?id=28526) | [eudicots](https://www.ncbi.nlm.nih.gov/Taxonomy/Browser/wwwtax.cgi?id=71240) | 36.2 | 1 |
| ..[Phaseolus vulgaris](https://www.ncbi.nlm.nih.gov/Taxonomy/Browser/wwwtax.cgi?id=3885) | [eudicots](https://www.ncbi.nlm.nih.gov/Taxonomy/Browser/wwwtax.cgi?id=71240) | 36.2 | 1 |
| ..[Perochaeta cuirassa](https://www.ncbi.nlm.nih.gov/Taxonomy/Browser/wwwtax.cgi?id=1411038) | [flies](https://www.ncbi.nlm.nih.gov/Taxonomy/Browser/wwwtax.cgi?id=7147) | 36.2 | 1 |
| ..[Candida tetrigidarum](https://www.ncbi.nlm.nih.gov/Taxonomy/Browser/wwwtax.cgi?id=434046) | [ascomycetes](https://www.ncbi.nlm.nih.gov/Taxonomy/Browser/wwwtax.cgi?id=4890) | 36.2 | 1 |
| ..[Octodon degus](https://www.ncbi.nlm.nih.gov/Taxonomy/Browser/wwwtax.cgi?id=10160) | [rodents](https://www.ncbi.nlm.nih.gov/Taxonomy/Browser/wwwtax.cgi?id=9989) | 36.2 | 1 |
| ..[Rhodosciadium argutum](https://www.ncbi.nlm.nih.gov/Taxonomy/Browser/wwwtax.cgi?id=40941) | [eudicots](https://www.ncbi.nlm.nih.gov/Taxonomy/Browser/wwwtax.cgi?id=71240) | 36.2 | 1 |
| ..[Ottoa oenanthoides var. oenanthoides](https://www.ncbi.nlm.nih.gov/Taxonomy/Browser/wwwtax.cgi?id=1128156) | [eudicots](https://www.ncbi.nlm.nih.gov/Taxonomy/Browser/wwwtax.cgi?id=71240) | 36.2 | 1 |
| ..[Myrrhidendron donnell-smithii](https://www.ncbi.nlm.nih.gov/Taxonomy/Browser/wwwtax.cgi?id=40924) | [eudicots](https://www.ncbi.nlm.nih.gov/Taxonomy/Browser/wwwtax.cgi?id=71240) | 36.2 | 1 |
| ..[Mathiasella bupleuroides](https://www.ncbi.nlm.nih.gov/Taxonomy/Browser/wwwtax.cgi?id=54722) | [eudicots](https://www.ncbi.nlm.nih.gov/Taxonomy/Browser/wwwtax.cgi?id=71240) | 36.2 | 1 |
| ..[Enantiophylla heydeana](https://www.ncbi.nlm.nih.gov/Taxonomy/Browser/wwwtax.cgi?id=40928) | [eudicots](https://www.ncbi.nlm.nih.gov/Taxonomy/Browser/wwwtax.cgi?id=71240) | 36.2 | 1 |
| ..[Arracacia ebracteata](https://www.ncbi.nlm.nih.gov/Taxonomy/Browser/wwwtax.cgi?id=884256) | [eudicots](https://www.ncbi.nlm.nih.gov/Taxonomy/Browser/wwwtax.cgi?id=71240) | 36.2 | 1 |
| ..[Aethusa cynapium](https://www.ncbi.nlm.nih.gov/Taxonomy/Browser/wwwtax.cgi?id=40954) | [eudicots](https://www.ncbi.nlm.nih.gov/Taxonomy/Browser/wwwtax.cgi?id=71240) | 36.2 | 1 |
| ..[Crithmum maritimum](https://www.ncbi.nlm.nih.gov/Taxonomy/Browser/wwwtax.cgi?id=40916) | [eudicots](https://www.ncbi.nlm.nih.gov/Taxonomy/Browser/wwwtax.cgi?id=71240) | 36.2 | 1 |
| ..[Mus musculus](https://www.ncbi.nlm.nih.gov/Taxonomy/Browser/wwwtax.cgi?id=10090) | [rodents](https://www.ncbi.nlm.nih.gov/Taxonomy/Browser/wwwtax.cgi?id=9989) | 36.2 | 12 |
| ..[Nitrosopumilus maritimus SCM1](https://www.ncbi.nlm.nih.gov/Taxonomy/Browser/wwwtax.cgi?id=436308) | [archaea](https://www.ncbi.nlm.nih.gov/Taxonomy/Browser/wwwtax.cgi?id=2157) | 36.2 | 1 |
| ..[Nematostella vectensis](https://www.ncbi.nlm.nih.gov/Taxonomy/Browser/wwwtax.cgi?id=45351) | [sea anemones](https://www.ncbi.nlm.nih.gov/Taxonomy/Browser/wwwtax.cgi?id=6103) | 36.2 | 1 |
| ..[Helobdella bolivianita](https://www.ncbi.nlm.nih.gov/Taxonomy/Browser/wwwtax.cgi?id=153510) | [segmented worms](https://www.ncbi.nlm.nih.gov/Taxonomy/Browser/wwwtax.cgi?id=6340) | 36.2 | 1 |
| ..[Brachypogon sp. BOLD-2016](https://www.ncbi.nlm.nih.gov/Taxonomy/Browser/wwwtax.cgi?id=1881749) | [flies](https://www.ncbi.nlm.nih.gov/Taxonomy/Browser/wwwtax.cgi?id=7147) | 30.2 | 2 |
| ..[Corynoptera subcavipes](https://www.ncbi.nlm.nih.gov/Taxonomy/Browser/wwwtax.cgi?id=1720653) | [flies](https://www.ncbi.nlm.nih.gov/Taxonomy/Browser/wwwtax.cgi?id=7147) | 30.2 | 1 |
| .[Cloning vector pME10](https://www.ncbi.nlm.nih.gov/Taxonomy/Browser/wwwtax.cgi?id=1770536) | [other sequences](https://www.ncbi.nlm.nih.gov/Taxonomy/Browser/wwwtax.cgi?id=28384) | 52.0 | 1 |
| .[S. aureus-E. coli shuttle vector pBUS1-Pcap-HC](https://www.ncbi.nlm.nih.gov/Taxonomy/Browser/wwwtax.cgi?id=1545807) | [other sequences](https://www.ncbi.nlm.nih.gov/Taxonomy/Browser/wwwtax.cgi?id=28384) | 52.0 | 1 |
| .[Expression vector pME9](https://www.ncbi.nlm.nih.gov/Taxonomy/Browser/wwwtax.cgi?id=1296589) | [other sequences](https://www.ncbi.nlm.nih.gov/Taxonomy/Browser/wwwtax.cgi?id=28384) | 52.0 | 1 |
| .[Shuttle vector pME8](https://www.ncbi.nlm.nih.gov/Taxonomy/Browser/wwwtax.cgi?id=1296590) | [other sequences](https://www.ncbi.nlm.nih.gov/Taxonomy/Browser/wwwtax.cgi?id=28384) | 52.0 | 1 |
| .[Expression vector pME10](https://www.ncbi.nlm.nih.gov/Taxonomy/Browser/wwwtax.cgi?id=1296588) | [other sequences](https://www.ncbi.nlm.nih.gov/Taxonomy/Browser/wwwtax.cgi?id=28384) | 52.0 | 1 |
| .[GFP expression vector pHapII](https://www.ncbi.nlm.nih.gov/Taxonomy/Browser/wwwtax.cgi?id=866769) | [other sequences](https://www.ncbi.nlm.nih.gov/Taxonomy/Browser/wwwtax.cgi?id=28384) | 52.0 | 1 |
| .[Shuttle vector pMK3](https://www.ncbi.nlm.nih.gov/Taxonomy/Browser/wwwtax.cgi?id=515189) | [other sequences](https://www.ncbi.nlm.nih.gov/Taxonomy/Browser/wwwtax.cgi?id=28384) | 52.0 | 1 |
| .[Shuttle expression-secretion vector pP43NMK](https://www.ncbi.nlm.nih.gov/Taxonomy/Browser/wwwtax.cgi?id=370428) | [other sequences](https://www.ncbi.nlm.nih.gov/Taxonomy/Browser/wwwtax.cgi?id=28384) | 52.0 | 1 |
| .[Cloning vector pND50](https://www.ncbi.nlm.nih.gov/Taxonomy/Browser/wwwtax.cgi?id=316466) | [other sequences](https://www.ncbi.nlm.nih.gov/Taxonomy/Browser/wwwtax.cgi?id=28384) | 52.0 | 1 |
| .[synthetic construct](https://www.ncbi.nlm.nih.gov/Taxonomy/Browser/wwwtax.cgi?id=32630) | [other sequences](https://www.ncbi.nlm.nih.gov/Taxonomy/Browser/wwwtax.cgi?id=28384) | 52.0 | 1 |
| .[Shuttle vector pHY320PLK](https://www.ncbi.nlm.nih.gov/Taxonomy/Browser/wwwtax.cgi?id=77677) | [other sequences](https://www.ncbi.nlm.nih.gov/Taxonomy/Browser/wwwtax.cgi?id=28384) | 52.0 | 1 |
| .[Plasmid pUB110](https://www.ncbi.nlm.nih.gov/Taxonomy/Browser/wwwtax.cgi?id=2655) | [other sequences](https://www.ncbi.nlm.nih.gov/Taxonomy/Browser/wwwtax.cgi?id=28384) | 52.0 | 2 |
| **.**[**Plasmid pMV158**](https://www.ncbi.nlm.nih.gov/Taxonomy/Browser/wwwtax.cgi?id=2606) | [**other sequences**](https://www.ncbi.nlm.nih.gov/Taxonomy/Browser/wwwtax.cgi?id=28384) | **52.0** | **2** |
| .[Clostridium phage CP3](https://www.ncbi.nlm.nih.gov/Taxonomy/Browser/wwwtax.cgi?id=2025812) | [viruses](https://www.ncbi.nlm.nih.gov/Taxonomy/Browser/wwwtax.cgi?id=10239) | 36.2 | 1 |
| .[Clostridium phage Clo-PEP-1](https://www.ncbi.nlm.nih.gov/Taxonomy/Browser/wwwtax.cgi?id=1927016) | [viruses](https://www.ncbi.nlm.nih.gov/Taxonomy/Browser/wwwtax.cgi?id=10239) | 36.2 | 1 |

Program: BLASTN 2.7.0+ (Zhang et al., 2004;Morgulis et al., 2008) Search database Nucleotide collection (nr/nt) using Megablast, on October 1^st^, 2017. Maximum number of aligned sequences to display: 500. Score 52.0 (100% identity).

**References**
